# Metabolomic Profiling of Post-Mortem Brain Reveals Changes in Amino Acid and Glucose Metabolism in Mental Illness Compared with Controls

**DOI:** 10.1016/j.csbj.2016.02.003

**Published:** 2016-02-26

**Authors:** Rong Zhang, Tong Zhang, Ali Muhsen Ali, Mohammed Al Washih, Benjamin Pickard, David G. Watson

**Affiliations:** a Strathclyde Institute of Pharmacy and Biomedical Sciences, 161, Cathedral Street, Glasgow G4 0RE, Scotland, UK; b Institute of Clinical Pharmacology, Guangzhou University of Chinese Medicine, No. 12 Jichang Road, Guangzhou 510405, China; c Department of Clinical Biochemistry/Diabetes and Endocrinology Centre, Thi-Qar Health Office, Thi-Qar, Nassiriya, Iraq; d General Directorate of Medical Services, Ministry of Interior, Riyadh 13321, KSA

**Keywords:** Metabolomics, Schizophrenia, Depression, Bipolar disorder, Diabetes, Brain tissue, Branched chain amino acids, Sorbitol

## Abstract

Metabolomic profiling was carried out on 53 post-mortem brain samples from subjects diagnosed with schizophrenia, depression, bipolar disorder (SDB), diabetes, and controls. Chromatography on a ZICpHILIC column was used with detection by Orbitrap mass spectrometry. Data extraction was carried out with m/z Mine 2.14 with metabolite searching against an in-house database. There was no clear discrimination between the controls and the SDB samples on the basis of a principal components analysis (PCA) model of 755 identified or putatively identified metabolites. Orthogonal partial least square discriminant analysis (OPLSDA) produced clear separation between 17 of the controls and 19 of the SDB samples (R2CUM 0.976, Q2 0.671, p-value of the cross-validated ANOVA score 0.0024). The most important metabolites producing discrimination were the lipophilic amino acids leucine/isoleucine, proline, methionine, phenylalanine, and tyrosine; the neurotransmitters GABA and NAAG and sugar metabolites sorbitol, gluconic acid, xylitol, ribitol, arabinotol, and erythritol. Eight samples from diabetic brains were analysed, six of which grouped with the SDB samples without compromising the model (R2 CUM 0.850, Q2 CUM 0.534, p-value for cross-validated ANOVA score 0.00087). There appears on the basis of this small sample set to be some commonality between metabolic perturbations resulting from diabetes and from SDB.

## 1 Introduction

Mental illness, most commonly schizophrenia, depression, and bipolar disorder (‘SDB’) is common: schizophrenia has a European prevalence of 0.2–2.6%, depression 3.1–10.1%, and bipolar disorder 0.2–1.1% [1]. These conditions are a major burden on the health-care systems and on the relatives of affected people. Clinically, such illnesses are heterogeneous and present with psychosis or mood state features that vary over time and across individuals. Thus, it would be of great value to have an objective means to assist in diagnosis and categorisation of such illnesses and also give an insight into the best way to manage them [2]. Much diagnosis of mental illness remains subjective due to complex and poorly defined mechanisms underlying these diseases; there are no biomarkers and mental illness might be better viewed as a continuum rather than using absolute labelling [3].The significance of low-molecular-weight metabolites in driving or reflecting the aetiology of psychiatric disease has been researched for many years using serum samples that are a pragmatic choice for diagnostic testing and, additionally, brain tissue to investigate the central pathologies [4], [5]. In the past 10 years, mass spectrometry-based metabolomics has evolved as a method for profiling a wide range of low-molecular-weight metabolites [6], [7]. Metabolomics is a natural fit with metabolite profiling in mental illness where, for many years, targeted analysis was carried out in order to profile, for instance, biogenic amines in order to determine whether or not abnormalities in their levels might be causative [8], [9]. There have been several studies which have carried out metabolomic profiling in mental illness [10], [11], [12], [13], [14], [15], [16], [17], but these have not been as extensive as those into other diseases such as cancer.

There have been no untargeted metabolomics studies of human post-mortem brain samples although there was a study which examined disturbed glucose metabolism in post-mortem brains from psychotic patients [18]. In the current study, the availability of a unique library of post-mortem brain samples with extensive associated medical information allowed us to investigate whether or not these samples might reveal any underlying pathology which could be related to metabolic differences. Thus, we applied our established LC-MS-based metabolomic profiling methods [19], [20] to determine if it was possible to individually classify healthy control, depressive, schizophrenic, and bipolar brains. The observation of ‘metabolic syndrome’-like features in those diagnosed with mental illness [21], [22] prompted us to determine whether or not there was an overlap between metabolic perturbations in mental illness and diabetes. If such a link between mental illness and diabetes could be established then this might give some rationale for the evaluation of medicines used in the treatment of diabetes in the treatment of mental illness.

## 2 Materials and Methods

### 2.1 Chemicals

HPLC-grade acetonitrile was obtained from Fisher Scientific, UK. Ammonium carbonate, ammonium hydroxide solution (28–30%), acetic anhydride, pyridine, and methanol were purchased from Sigma–Aldrich, UK. HPLC grade water was produced by a Direct-Q 3 Ultrapure Water System from Millipore, UK. The mixtures of metabolite authentic standards were prepared as previously described [19], [23] from standards obtained from Sigma–Aldrich, UK.

### 2.2 Post-Mortem Brain Samples

Post-mortem brain samples were obtained from the Sudden Death Bank collection held in the MRC Edinburgh Brain and Tissue Banks. Psychiatric diagnosis annotations for each sample were made by detailed study of donor case notes by qualified psychiatrists. Full ethics permission has been granted to the Banks for collection of samples and distribution to approved researchers (LREC 2003/8/37). The University of Strathclyde Ethics Committee also approved the local study of this material (UEC101123). Details of the brain samples are given in Table A1 in the Appendix. The information regarding the brain samples is summarised in Table 1.

### 2.3 Sample Extraction

The brain samples were thawed and then a sample of brain tissue (50 mg) was homogenised in ice cold methanol/water (1:1, 1.5 ml) using a handheld Lab Gen 7B homogeniser. The samples were then centrifuged at 16,000*g*, 15 min 4 °C and the supernatant was removed and the pellet reserved for further extraction to remove lipids. Lipids were extracted from the pellet with chloroform/methanol (3:1, 1.6 ml). The methanol/water extract was dried under a stream of nitrogen at 37 °C and redissolved in acetonitrile/water (80:20, 200 μl), the sample was centrifuged 16,000*g*, 15 min 4 °C to remove any insoluble material and then analysed by ZICHILIC and ZICpHILIC chromatography. The chloroform/methanol extract was dried under a stream of nitrogen at 37 °C and re-dissolved in either methanol/water (1:1, 200 μl) or methanol/chloroform (1:1, 200 μl) prior to chromatography on either C18 column or silica gel, respectively.

### 2.4 HILIC–HRMS Analysis

Sample analysis was carried out on an Accela 600 HPLC system combined with an Exactive (Orbitrap) mass spectrometer (Thermo Fisher Scientific, UK). An aliquot of each sample solution (10 μl) was injected onto a ZIC-pHILIC column (150 × 4.6 mm, 5 μm; HiChrom, Reading UK) with mobile phase A: 20 mM ammonium carbonate in HPLC grade water (pH 9.2), and B: HPLC grade acetonitrile. The LC and the MS conditions were as described previously [19], [20]. Samples were submitted in random order for LC-MS analysis, and pooled quality control samples were injected at the beginning, middle, and end of the experiment to monitor the stability of the instrumentation. Standard mixtures containing authentic standards for 220 compounds were run in order to calibrate the column. Further analysis of the polar extract and of the lipophilic extracts were carried out on a ZICHILIC column (150 × 4.6 mm, 5 μm), ACE C18 column (150 × 3 mm, 3 μm), and an ACE silica gel column according to our previously described methods [23], [24].

### 2.5 Analysis of Sugar Acids and Polyols by GC-MS

The individual standards for the polyols (100 μg) were treated with acetic anhydride/pyridine (1:1, 100 μl) for 30 min at 70 °C. The reagent was removed under a stream of nitrogen and the sample was re-dissolved in ethyl acetate 1 ml. The individual standards for the polyol acids were treated with methanol containing 1% HCl for 30 min at 70 °C, the reagent was removed under a stream of nitrogen and the sample was then treated as for the polyols. Brain tissue (200 mg) was extracted with acetonitrile/water (1:1, 1 ml) containing 2 μg/ml of pinitol internal standard, centrifuged and the supernatant was removed and evaporated to dryness with a stream of nitrogen at 70 °C and treated as for the polyol acids except that the residue was re-dissolved in 0.2 ml of ethyl acetate. GC-MS analysis was carried out on a DSQ GC-MS system (Thermo Fisher Scientific, UK) fitted with a GL Sciences Inert Cap 1 MS column from Hichrom, Reading UK(30 m × 0.25 mm × 0.25 μm film). The oven was programmed from 100 °C to 320 °C at 5 °C/min. The MS was operated in EI mode at 70 eV. For quantification of the sugars in brains selected, ion monitoring was carried out for ions at m/z 217, 200, 187, 145, 142, and 140, which are typical fragments of alditol acetates [25].

### 2.6 Data Extraction and Metabolite Identification

MZMine 2.14 [26] was used for peak extraction and alignment, as previously described [19], [20]. Putative identification of metabolites was also conducted in MZMine by searching the accurate mass against our in-house database [18], [19], [23]. Background peaks present in the blank were removed in MZmine before transferring the data to an Excel file. Manual editing of the data was carried out in order to remove idiosyncratic peaks such as metabolites identified as drugs which were presumably from patient treatments and also nicotine metabolites which were particularly abundant in the brains of schizophrenic patients because of their well-established tendency to smoke much more than the general population [27] and ethyl sulphate which is from alcohol metabolism. The GC-MS data were extracted by using Sieve 1.3 (ThermoFisher Scientific UK), and the ions corresponding to the retention time of the sugar standards were extracted in order to build the OPLS-DA model.

### 2.7 Multivariate and Univariate Analysis

All data processing, including data visualisation, biomarker identification, diagnostics, and validation was implemented using SIMCA software v.14 (Umetrics AB, Umeå, Sweden). Prior to multivariate analysis, data were pareto scaled where the responses for each variable are centred by subtracting its mean value and then dividing by the square root of its standard deviation [28], [29]. Principal component analysis (PCA) was used to provide an unsupervised model in order to explore how variables clustered regardless *Y* class [30]. Orthogonal projections to latent structures (OPLS) provides a supervised model that can predict *Y* from *X* and can separate variation in *X* that correlates to *Y* (predictive) and variation in *X* that is uncorrelated to *Y* (orthogonal/systemic). OPLS-DA is a discriminant analysis based on OPLS and employed to examine the difference between groups while neglecting the systemic variation [30]. The p-values of the biomarkers were evaluated for their significance applying the false discovery rate statistic (FDR) [31]. Variable importance in the projection (VIP) was employed in order to indicate the contribution of each variable in the in a given model compared to the rest of variables [32], the average VIP is equal to 1, based on that a variable larger than 1 has more contribution in explaining *y* than the average [33].

### 2.8 Diagnostics and Validation of Models

R^2^ and Q^2^ are diagnostic tools for supervised and unsupervised models; R^2^ represents the percentage of variation explained by the model (the goodness of fit), Q^2^ indicates the predictive ability of the model [34], [35], [36], a large discrepancy between between R^2^ and Q^2^ indicates overfitting of the model. A permutations test can be applied to supervised models to evaluate whether the specific grouping of the observations in the two designated classes is significantly better than any other random grouping in two arbitrary classes [34], [35], [36], and in Simca P, this is carried out by repeatedly leaving out 1/7th of the data an refitting the model, all the Q^2^ values for the refitted models should be lower than the original Q^2^ value. The criteria for validity for OPLSDA models tested via cross-validation are that all blue Q^2^-values to the left are lower than the original points to the right or the blue regression line of the Q2-points intersects the vertical axis (on the left) at, or below zero. The R^2^ values always show some degree of optimism. However, when all green R^2^-values to the left are lower than the original point to the right, this is also an indication for the validity of the original model although this is not essential for the model to be valid. Model validity is also assessed using cross-validated ANOVA (CV-ANOVA) which corresponds to H_0_ hypothesis of equal cross-validated predictive residuals of the supervised model in comparison with the variation around the mean [37]. Univariate comparisons were carried out in Excel.

## 3 Results

### 3.1 The Effect of the HPLC Column Used on the Results

The data produced from the analysis of the polar extracts on the ZICHILIC column were less satisfactory for producing separation in the sample sets than those produced on ZICpHILIC. There were similar trends in some of the metabolites but the clear-cut differences described below were not observed. This again supports our choice of ZICpHILIC as the best method for analysis of polar metabolites in metabolomics screens [13]. The ZICHILIC mobile phase produces a higher background which includes abundant sodium formate cluster ions and thus ion-suppression is potentially more of a problem. In addition, the chromatographic peaks for many metabolites are wide than om ZICpHILIC and the retention times from run to run are less stable which produces a greater challenge for the peak extraction software. The lipid fractions were analysed on silica gel and C18 columns and no major differences in lipid profiles were observed between the controls and the SDB brains. This may be due in part to the fact that the initial methanol/water extraction also extracted many of the more polar lipids. The chromatography of lipids on the ZICpHILIC column is satisfactory but they are only weakly retained on this column so there is no separation of isomeric species.

### 3.2 Comparison of Control and Schizophrenic/Depressive/Bipolar/Diabetic Brains using PCA

**Fig. 1:**
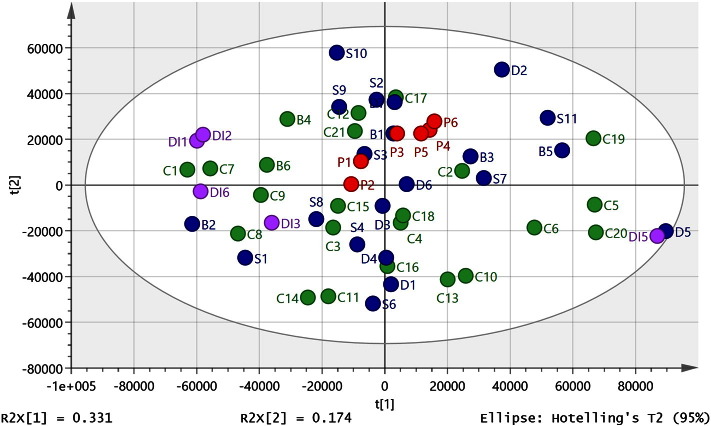
PCA plot for control, SDB and DI samples (R^2^X cum 0.61, Q^2^ (cum) 0.464, 3 components) based on 755 metabolites from positive and negative ion modes. Three of the DI samples (DI4, 7 and 8) lay outside of the ellipse and were omitted from the model. P = pooled sample used to check instrument stability over time.

Metabolites were identified to MSI levels 2 or 3 [38] according to either exact mass (< 3 ppm deviation) or exact mass plus retention time matching to a standard. After data filtering, 755 metabolites from positive and negative ion modes were combined and used to build multivariate models. The sample set was selected by our collaborators at the sudden death brain and tissue bank to give us the best sample set available from samples in storage for making a comparison between controls, mental illness, and diabetes. Since the uncontrolled factors are highly variable in both control and affected samples, the expected result might be that variation in the data would preclude statistical separation unless the disease signature was very strong. In order to obtain a reasonable sample size, we treated schizophrenic, depression, bipolar (SDB), and diabetic (DI) samples as one group to compare against controls. Comparison of the data from schizophrenic, depression, bipolar (SDB), and diabetic (DI) samples and controls using PCA did not yield a clear separation of these diagnostic categories (Fig. 1). In order to rule out variation in level of technical precision across the *ca* 55 h required to complete the analysis, a pooled sample: (P1–6) was prepared by combining 5 × 40μl of extract randomly selected from each sample type. Replicates were run as follows: P1 and 2 near the beginning of the sequence after running three blanks and four standard mixtures, P3 after *ca* 20 h, P5 after *ca* 39 h, P4 and 6 at the end of the run after *ca* 55 h. As can be seen in Fig. 1, the pooled samples all lie towards the centre of the PCA plot and individual sample points are close to each other. This indicates that there is only a small amount of instrumental drift and thus the results reflect biological, rather than technical, differences.

### 3.3 Comparison of SDB and SDBDI Samples Against Controls Using OPLSDA

**Fig. 2:**
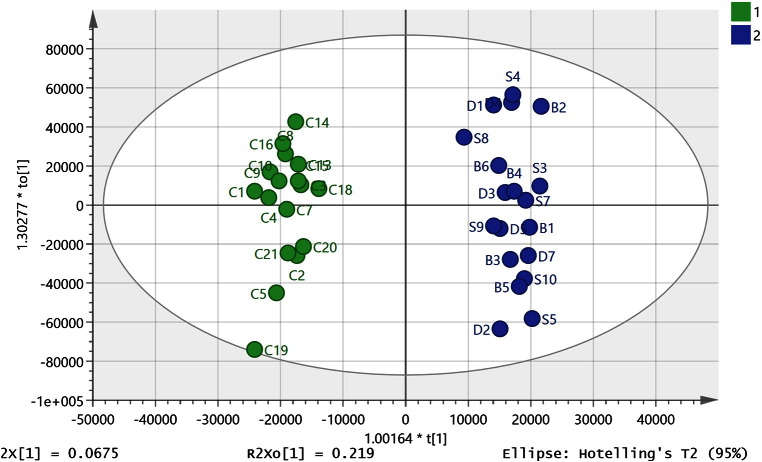
OPLS-DA model (R^2^CUM 0.976, Q^2^CUM 0.671, 6 components) of control (n = 17) compared to SDB (n = 19) brain samples based on 755 metabolites from positive and negative ion modes.

When the DI samples were omitted, it was found that 36 of the 44 available SDB and control samples (see footnote to Table A1) could be combined where 19 SDB samples were compared against 17 control samples to produce a strong OPLS-DA model (Fig. 2) (R^2^CUM 0.976, Q^2^CUM 0.671) explaining 96.7% of the variation in the samples with six components. Q2 > 0.5 is generally accepted as being indicative of a robust model [35], [36] and the model gave a permutations plot where all the permutated Q^2^ values (n = 999) on the left are lower than the points on the right (Figure A 1) and the line plot intercepts the y-axis below 0 [34], [35], [36]. This preliminary model was used to inform the selection of the samples for univariate statistical comparison by excluding 8 samples that did not fit the model, four controls and four SDB samples. Despite variations arising from complex medical histories, length of sample storage and exact cause of death there appeared to be a strong metabolic signature associated with mental illness overriding these confounding factors which apply to both control and affected samples. Table 1 also shows the univariate statistical comparisons for the metabolites with VIP scores > 1 in the preliminary OPLSDA model. All of which are significantly different according to a two-tailed t-test and FDR statistics [31] based on 755 metabolites indicate all P values < 0.05 are significant. A complete list of significantly different metabolites based on univariate comparison of the 17 controls and 19 SDB samples is given in Table A2. The initial application of OPLSDA based on 755 metabolites allowed us to focus on more limited list of metabolites than those listed in table A2.

### 3.4 SDB Samples Show Differences in Branched Amino Acid, Neurotransmitter and Sugar Metabolism Compared With Controls.

Leucine/isoleucine have the highest VIP (8.1) this a very strong variable along with valine which has a VIP of 5.1. Thus, branched chain amino acids are highly correlated in the brains of SDB subjects and are also present in significantly higher levels than in the controls. The other neutral lipophilic amino acids methionine, phenylalanine, tyrosine, tryptophan, and proline also have high VIP values (Table 2 and table A2), are elevated in SDB brains and are important in the model. The important metabolites that are significantly lower in the SDB subjects than in the controls include GABA, its metabolite guanidino amino butyric acid, and the neuromodulator N-acetyl aspartyl glutamate (NAAG). In addition, there are higher levels of sugar metabolites, putatively identified according to the LC-MS analysis as sorbitol, gluconic acid, and erythritol, in the SDB samples.

### 3.5 The Effect of Age on Metabolite Profiles of Brain Tissue

The brains were from subjects with a wide age range and the mean age of the control group at death was 45.9 and mean age for the SDB group was 46.4. An OPLS model (R^2^X (cum) 0.706), R^2^Y (cum) 0.979, Q^2^ (cum) 0.476)) gave a very good correlation between age and metabolites (Figure A2) and there was no overlap between the metabolites used to discriminate the control and SDB brains and those which discriminated age (Table A3). The major changes with age were related to decreases in unsaturated fatty acids in the brain such as eicosatetraenoic, docosahexaenoic, and linoleic acid and increases in glycerol metabolites such as phosphoethanolamine and phosphocholine.

### 3.6 Inclusion of Diabetic Brains in the OPLSDA Model

**Fig. 3:**
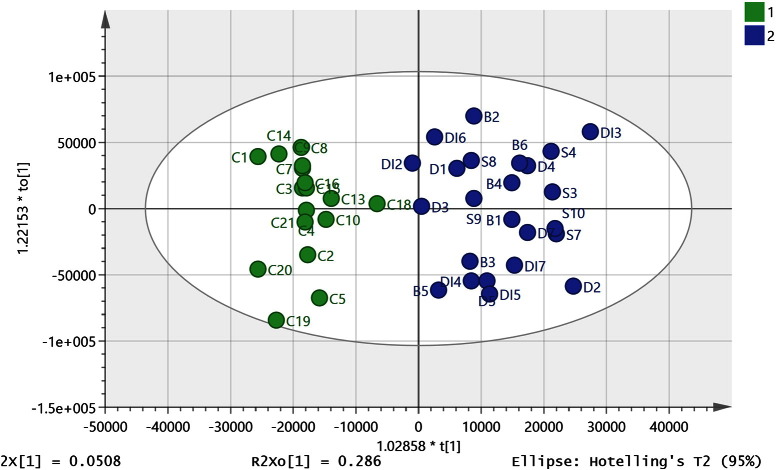
OPLS-DA model (R^2^ (cum) 0.850, Q^2^ (cum) 0.534, 4 components) including six of the DI samples. Green control and blue SDB + diabetic brain samples based on 755 metabolites from positive and negative ion modes.

There is evidence that there may be some shared pathology between diabetes and mental illness going back as far the pre-neuroleptic drug era and this provided the rationale for the insulin coma therapy which was used in the first half of the 20th century [39]. There were eight diabetes samples in the set of brain samples and these were subsequently added to the data set used to build the OPLSDA model described above. Two of the diabetic samples (DI1 and DI8) were extreme outliers and were excluded from the initial PCA plot (Fig. 1) since they were outside of the ellipse. They were also excluded from the combined OPLSDA model along with one of the SDB samples (S5) which was excluded since it did not fit into the new OPLSDA model. Six of the diabetic samples could be classified with the SDB samples (Fig. 3, R^2^ CUM 0.850, Q^2^ CUM 0.534, p-value for cross-validated ANOVA score 0.00087) increasing the significance of the ANOVA score, the large decrease in the CVANOVA score implies considerable strengthening of the model since the score can be used as a guide to the optimal fitting of a model [36]. The permutations plot is shown in Figure A3 indicates a strong model. The addition of the diabetic samples to the model produced some change in the VIP values but basically most of the discriminating metabolites are the same (Table A3) which is perhaps not surprising since the model is strengthened by addition of these samples. However, when the univariate comparisons are examined most of the metabolites with high VIP values in the model did not have significant p-values when the diabetic samples in the model are compared with controls. Thus, the similarities between diabetic and the SDB brains lie in the covariance of the set of important marker compounds shown in Table A3 rather than in the absolute levels. Leaving the diabetic samples out of the model and using them as a prediction set resulted in four of the samples being classified as SDB samples while two were unclassified but borderline to the SDB class.

### 3.7 Preparation of PCA Model with a Reduced Metabolite List

**Fig. 4:**
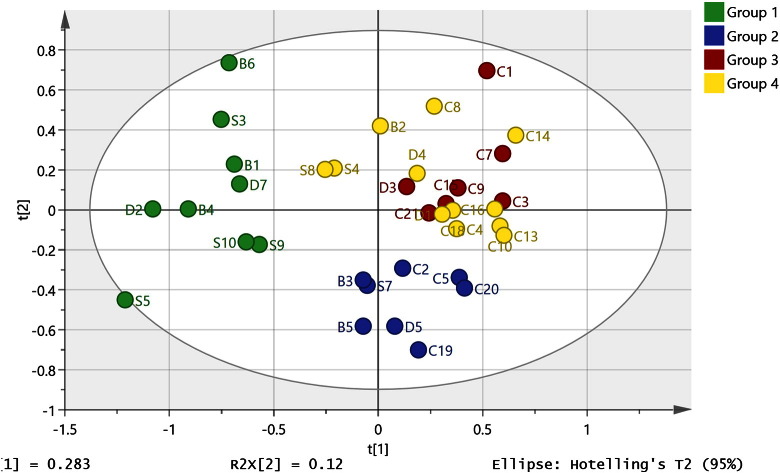
PCA model ( R^2^X = 0.68, Q^2^X = 0.283, 5 components) based on the metabolites with P values < 0.05 when the control and SDB samples used to prepare the OPLS-DA model shown in Fig. 2 are compared and hierarchical cluster analysis is used to define subgroups. The analysis reveals a clear subgroup (group 1) containing depressive/bipolar and schizophrenic samples which is distinctly different from the rest of the samples.

When the reduced list of 120 metabolites with low P values shown in Table A2 was used to prepare a PCA model, it was clear that the SDB samples contained subgroups. In particular, a group of nine SDB samples were quite different from the controls and the rest of the SDB samples as shown in Fig. 4 where HCA was used to define the groups (HCA tree shown in Figure A5). The metabolites defining the subgroup are shown in Table 3. This supports the proposal that there are similarities within the SDB group since the sub-group contains all three classes.

### 3.8 Refining the OPLSDA Models

**Fig. 5:**
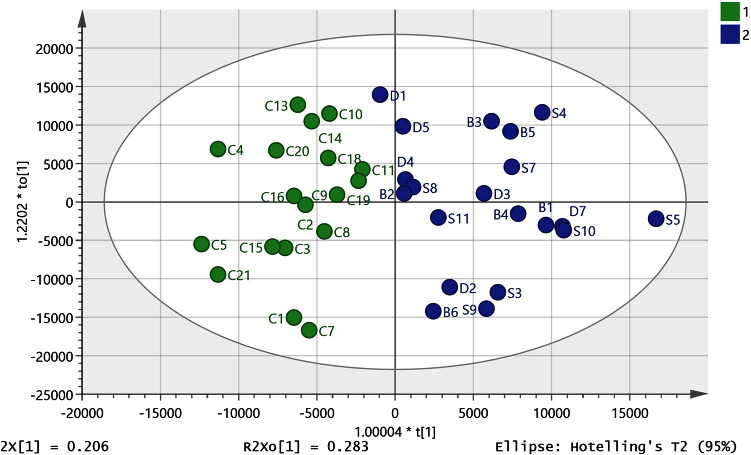
OPLSDA (R^2^Y cum 0.725, Q^2^ cum 0.638, five components) model based on the six metabolites shown in Table 4 classifying 17 controls (1) and 21 SDB samples (2).

**Fig. 6:**
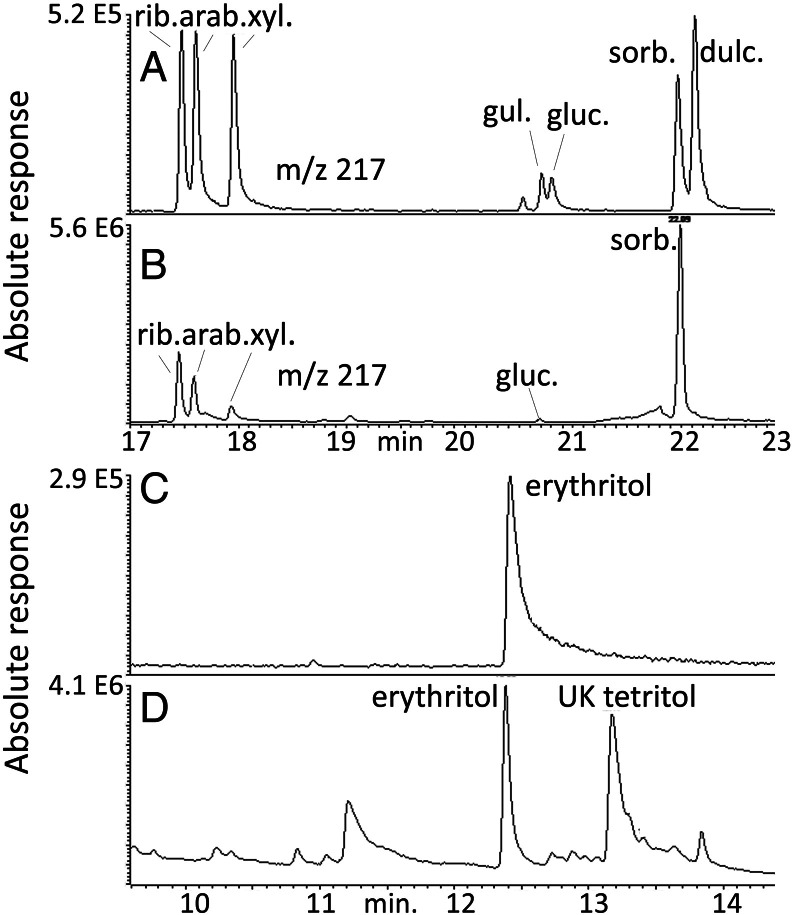
OPLSDA model (R^2^Y cum 0.798, Q^2^ cum 0.691, 4 components) based on the six metabolites shown in Table 4 classifying 18 controls (1) and 27 SDBDI samples (2).

The purpose of this study to try to better understand disease pathology in mental illness and thus the ideal outcome would be a list of related metabolites corresponding to the disease state in order to develop a hypothesis. Of lower priority was to provide a classification system in the current case since sampling brain tissue is not going to be a diagnostic test. With a high number of variables, there is the danger of overfitting and although the OPLSDA models shown in Fig. 2, Fig. 3 performed well in cross-validation tests, there might still be some doubts with regard to their validity. Thus, the control/SDB OPLSDA model was refined by removing 600 of the lowest priority variables and then systematically removing variables one at a time from the remaining set while retaining variables that caused a reduction in the Q cum score of > 0.05 when removed. This resulted in the model shown in Fig. 5 which had a CVANOVA score of 0.0006 and which could accommodate 38 out of the original 44 samples based on the six metabolites shown in Table 4. The cross-validation model is shown in Figure A5. Removal of samples belonging to each sub-group in order to create prediction sets gave the results shown in Table 5. This resulted in two out of 21 subjects being misclassified. The sample size is relatively small so removing in each case around 15% of the samples will considerably weaken the model. In reduced model, the branched chain amino acid valine and the neurotransmitter GABA retain their high importance. The same process of variable reduction was applied to the combined diabetic/SDB model which included six of the diabetic samples and resulted in a model based on six metabolites into which 45 out of 53 samples could be fitted and included seven of the diabetic samples (Fig. 6) and which had a CVANOVA score of 0.000013. The metabolites included in the model were the same as those used in the model shown in Fig. 5 except that the VIP values for each metabolite were different (Table 6). Removing the 7 diabetic samples and using them as a prediction set resulted in six of the samples being classified with the SDB group and one of the samples being classified with the controls (details shown in Table 5).

### 3.9 GC-MS Analysis to Quantify and Identify Polyols and Polyol Acids in Brain

**Fig. 7:**
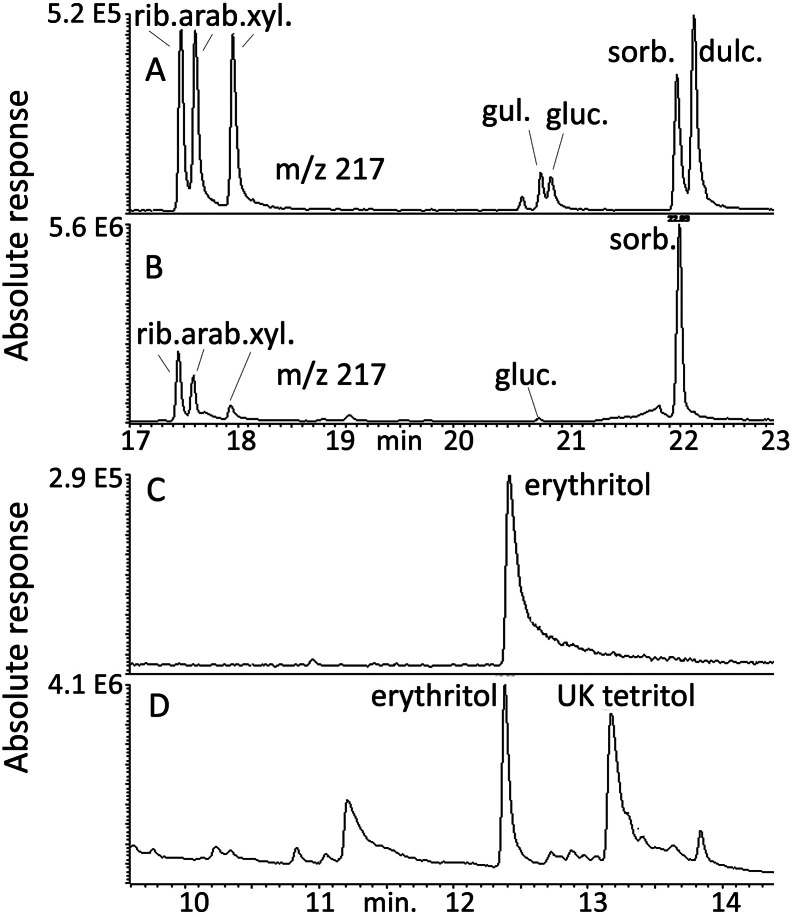
GC-MS analysis of polyol standards (A and C 0.8 μg/0.2 ml) in comparison with polyols in brain (B and D).

In the process of analysing the data, it became apparent that sugar metabolism had an important role in distinguishing the control and SDB brains. The commercially available sugar alcohol standards were run on the ZICpHILIC column and isomeric compounds were found to co-elute or elute closely and thus were not distinguishable from each other. A GC-MS method was developed for the analysis of the sugar alcohols which were converted into their acetates after initial treatment with methanolic HCl to esterify the acidic groups in gluconic and gulonic acid. The retention times for the available standards are shown in Table A5. Fig. 7 shows the separation of some the polyols present in brain tissue in comparison with a mixture of standards. The major polyols present were erythritol plus an additional unknown tetritol, ribitol, arabinotol, xylitol, gluconic acid, and sorbitol (Fig. 7). Figure A7 shows OPLS-DA separation of control and SDB + diabetic samples based on the ions monitored for the polyol standards; there was not sufficient tissue to repeat analysis of all the samples and the model is based on 21 SDBDI samples compared to 15 control samples. Figure A8 shows the cross-validation for the model indicating the there was a robust discrimination. Calibration curves were prepared in the range 1–16 μg for all the sugar alcohols against 2 μg of pinitol which was used as an internal standard. The data for the calibration curves are shown in Table S4. The quantitative data for the sugar alcohols are shown in Table 7. Clearly, both the diabetic samples and SDB have high levels of sorbitol, gluconic acid, ribitol, and erythritol in comparison to the controls. Elevated levels of sorbitol in schizophrenic and bipolar brains have been reported before [18], but the addition of the other sugar metabolites re-enforces the importance of this pathway in the illness.

## 4 Discussion

### 4.1 High Levels of BCAs and Other Liphilic Amino Acids in SDB Samples

The OPLSDA models based on the larger number of variables (Fig. 2, Fig. 3) and the univariate differences will be used to develop some hypotheses based on the underlying metabolite differences. The highest VIPs in the OPLS-DA model (Fig. 2) of the SDB samples against the controls are the branched chain amino acids leucine/isoleucine and valine (BCAs) which are elevated above the levels found in the controls. The importance of these metabolites in schizophrenia and bipolar disorder has recently been highlighted [40]. There have been a number of recent metabolomics studies of obesity and insulin resistance and it has been observed that there is a distinct metabolic signature linked to metabolic syndrome where the plasma levels of branched chain amino acids (BCAs) leucine, isoleucine, and valine were elevated together with methionine, glutamine, phenylalanine, tyrosine, asparagine, and arginine [41], [42]. A study which was carried out on a cohort of 1872 individuals who were subdivided in lean, overweight, and obese groups proposed that BCA levels can provide a better signature of metabolic wellness than BMI [43]. Another group found that elevated levels of BCAs in plasma could be linked to obesity and potentially to the development of insulin resistance in children and adolescents [44]. BCAs are known to promote production of muscle protein [45], [46] and an elevation in BCA levels may indicate that the uptake of BCAs into muscle tissues is reduced. Metabolomic profiling of plasma from schizophrenics, even before medication, has been found to indicate that they are at risk of developing metabolic syndrome [47]. Antipsychotic medications are known to significantly increase metabolic complications and induce weight gain and although the medication history of the relating to current samples is unknown it unlikely that variations in medication alone would be sufficiently systematic to account for the differences observed. In addition to BCAs, the neutral amino acids proline, methionine, tyrosine, and tryptophan are all elevated in the SDB/diabetic group and have high VIP values. There is an early report of marginal differences in the levels of lipophilic amino acids in plasma from schizophrenics with valine, phenylalanine, alanine, leucine, isoleucine, methionine, and tyrosine all being elevated [48]. However, the current data do not support the theory proposed by that paper, which was that elevation in lipophilic plasma acids might produce competition for lipophilic amino acid transporters into the CNS, resulting in reduced uptake of the amino acids tyrosine and tryptophan which are required for neurotransmitter biosynthesis.

Proline is a potential precursor of glutamate which is a neurotransmitter in brain and it has previously been shown to be increased in individuals diagnosed with schizophrenia. There is an extensive literature indicating that proline dehydrogenase (PRODH) activity may be up-regulated in schizophrenia [49], [50]. However, this would be expected to lead to a fall in proline levels which does not fit with the current observation.

### 4.2 Alterations in Sugar Metabolism.

It was not possible to clearly identify the different sugar alcohols using LC-MS since the isomeric compounds have almost identical retention times and their MS/MS spectra are very similar. In order to get a clearer identification of the sugar alcohols in brain, standards and a brain extracts were derivatised and analysed by GC-MS. The high chromatographic resolving power of a capillary GC column was able to separate the isomers. The levels of glucose in these brains appeared to be very low and the major sugar in the brain was myo-inositol. As can be seen in Fig. 7, there are several sugar alcohols in the brain. The presence of these compounds has been observed before in human CSF [51] where has been proposed that the likely source of the polyols was from the metabolic activity of the brain. The major hexitol in the brains is sorbitol but the pentitol peak observed in LC-MS as a single peak is due to the presence of three compounds, ribitol, arabinotol, and xylitol. In addition, there are two tetritols, erythritol, and an unknown isomer which are also elevated. It has been observed that the levels of these polyols in brain are elevated in response to osmotic stress [52]. In the current case, the levels of sorbitol, gluconic acid, ribitol, and erythritol are higher in the SDB/diabetic samples in comparison with the controls as judged from both the LC-MS and the GC-MS data (Table 3).

### 4.3 Elevation of Polyols and Oxidative Stress

Since the sugar alcohols are not closely linked within a particular pathway and several are elevated this suggests that the higher levels might be due to an upregulation of aldose reductase which has a wide substrate specificity [53] and is able reduce many different aldoses. Formation of sugar alcohols via aldose reductase activity is responsible for some of the complications of diabetes [53] and also generates oxidative stress since NADPH is consumed in carrying out the reduction. A previous paper observed that altered glucose metabolism is the brains of those diagnosed with depression and schizophrenia, where sorbitol was increased by a factor of 2.2 [18], similar to the elevations in the SDB brains in the current study. A recent metabolomic study observed altered glucose metabolism in peripheral blood mononuclear cells in schizophrenia with alterations in several glycolysis and Krebs cycle metabolites [11]. Glucaric acid, which is increased in SDB, also contributes to the model and has a high correlation to the model. Glucaric acid is of interest since it also relates to sorbitol and gluconic acid, being only a single oxidation step away from gluconic acid. Glucaric acid has been frequently monitored in urine as a marker of xenobiotic stress and urinary levels have been observed to rise in response to treatments with phenothiazines (such as the antipsychotic, chlorpromazine) [54]. Sedoheptulose is considerably elevated in SDB brains while a compound putatively identified as deoxysedoheptulose phosphate is depressed. Our published metabolomics study of brain tissue from a mouse model of psychiatric disorder, the *Npas3* knockout, also showed elevated levels of sedoheptulose (2.65-fold increase) [55]. This suggests that changes in glycolysis or nucleotide metabolism might be altering flux through the pentose phosphate pathway which could correlate with an increased requirement for NADPH as a co-factor for aldose reductase since NADP is converted to NADPH with formation of phosphogluconate at the entry to the pentose phosphate pathway. In addition, there is a deficit in the neuromodulator NAAG in the SDB brains; NAAG has been found to protect against neuronal death induced by exposure to glucose in a cell-culture model of diabetic neuropathy [56].

### 4.4 GABA Deficiency

GABA and its metabolite guanidino butyrate, which is formed directly from GABA via arginine–glycine amidinotransferase [57] are important variables in the OPLS-DA model separating control and SDB brains. It is well established that there is a deficit in GABAergic transmission in schizophrenia [58], [59], [60]. The GABA receptor governs the entry of the chloride ion into cells [61] and one of the highest VIP values in Table 1 is for an adduct formed between chloride and carbonate which is present in the mobile phase which is strongly correlated with the SDB group. Initially, this peak was assigned to orthophosphate according to the library search but it runs earlier than the standard for orthophosphate. Inspection of the peak revealed a chlorine isotope pattern and the elemental composition matches the carbonic acid/chloride adduct. Chloride itself is below the lower mass range cut off for the instrument.

### 4.5 Altered Purine Metabolism

From the univariate comparisons, the purines guanine and guanosine were found to be lower in SDB brains and this can be correlated with elevated levels of uric acid in SDB brains. In a recent publication, it was observed that the severity of schizophrenic symptoms could be predicted from a high ratio of uric acid to guanine and the current data indicate that in SDB brains purine oxidation seems to be more active [62]. It has been suggested that that elevated uric acid is indicative of high levels of oxidative stress. Allopurinol, which inhibits purine oxidation, has been used as an experimental treatment for schizophrenia [63].

### 4.6 Elevation in the Level of a Homocarnosine Isomer

A compound, present in high amount in the brains, putatively identified as anserine since it is isomeric with homocarnosine but has a different retention time, is an important component is the OPLS-DA model separating control and SDB brains and it is significantly lower in the SDB samples according to the univariate data. Brain tissue accounts for around 20% of the oxygen consumption by the body and thus is a major site of oxidative stress. Carnosine, homocarnosine, and anserine are important antioxidants in brain and skeletal muscle [64] and lower levels of anserine might indicate increased oxidative stress in the SDB samples. Of the three commonly occurring histidine dipeptides, anserine has been observed to be the most effective anti-oxidant [64].

### 4.7 High Levels of Pyridoxine

The SDB brains contain higher levels of pyridoxine which has been used for many years as an experimental treatment for schizophrenia when given in conjunction with nicotinic acid [65].

### 4.8 Alterations in Biogenic Amine Metabolism

A number of neurochemically important compounds are significantly changed in the univariate data. Tryptamine has long been associated with mental illness particularly schizophrenia [8] and it is clearly slightly elevated in the SDB brain samples. In the SDB samples, there is a depression of norepinephrine sulphate levels; there are two isomers of this compound in brain both of which are depressed. Glucuronide and sulphate conjugates of dopamine and serotonin have been measured in brain dialysate previously [66]; there was no evidence in the current case for the presence norephinephrine glucuronide conjugates in brain. Although not significant in the OPLS-DA model, in the univariate analysis, N-acetylvanilalanine is significantly elevated in bipolar brains. This metabolite is of great interest since it is a marker for a deficiency of aromatic amino decarboxylase (AADC/DOPA Decarboxylase) deficiency which can lead to a deficit in the levels of several neurotransmitters [67]. This can be correlated with elevated levels of all the aromatic amino acids in the SDB brains. There are also elevated levels of kynurenine and kynurenamine which are metabolites of tryptophan which have neuropathological effects [68].

### 4.9 Differences in a Sub-group of SDB Samples

There are many other differences in the univariate data and it is difficult to rationalise them all. In order to determine if there are subgroups within the samples, a PCA model was fitted to the 36 samples used to produce the OPLS-DA model using only the metabolites shown in Table S1 which were significantly different according to univariate analysis. Hierarchical cluster analysis clearly highlighted a group of 9 SDB samples which were far away from the rest of the samples that did not clearly separate in the PCA plot (Fig. 4). The metabolites which were most significant in separating this sub-group from the controls in the PCA plot are listed in Table 3 along with P values and ratios derived from univariate comparison of these nine samples with the control samples. The brains in this subgroup contain much lower levels of NAAG in comparison with the rest of the SDB group and sorbitol, gluconic acid, and xylitol/ribitol/arabinotol are also higher than in the general cohort. In addition, N-acetylvanilalanine is higher in these samples along with tyrosine and phenylalanine than in the rest of the SDB samples which might indicate a greater degree of aromatic amino acid decarboxylase deficiency. However, tryptophan is not significantly different in this subgroup compared with the rest of the SDB samples although its metabolite tryptamine is elevated. In addition norepinephrine sulphate and guanosine are significantly lower in this group compared with the rest of the samples.

### 4.10 OPLSDA Models Based on Six Metabolites

Figures A10–14 show extracted ion traces for the six marker compounds used to produce the OPLSDA models shown in Fig. 5, Fig. 6. GABA and valine are important components in the models shown in Fig. 2, Fig. 3 and have been discussed above. Ascorbic acid does not show up strongly with regard to univariate statistics having a P value of 0.5 when comparing the samples modelled in Fig. 2. There have been reports of increased ascorbic acid requirements in schizophrenia with reduced urinary excretion being observed [69]. Carnitine and its acyl derivatives have been reported to have potential in the treatment of neurochemical disorders [70]. Finally, it was observed in a previous study that pyruvate levels were lowered in the thalmus of the post-mortem brains of schizophrenics in comparison with controls [71]. In the current case, pyruvate is slightly elevated in the SDB group but the part of the brain analysed in the current case was different.

## 5 Conclusion

In conclusion, many differences were observed in SDB versus control brains which have been observed by previous papers such as lower levels of NAAG and GABA in the SDB brains, elevated levels of sorbitol, and the importance of branched chain amino acids. Our strategy of treating the three psychiatric disorders as a single disease entity (SDB) may reduce the ability to detect specific aetiological biomarkers, but it increased sample size, diluted disease-specific medication effects, and most importantly, allowed the identification of a metabolic profile reflecting a shared pathological state. Since there are no biomarkers for mental illnesses and these diseases are multifaceted, diagnosis is never absolute and indeed this can be seen in scatter plots where each individual is different. However, there is enough in common in the SDB group for them to be classified as more similar to each other than to the controls. Certain key metabolites highlighted as being more important in the pathology and it seems that abnormal sugar and branched chain amino acid metabolism might be a key element in SDB as reflected in the metabolic similarity between SDB and diabetes, and thus anti-diabetic treatments might have a role in the management of SDB. There are no previous metabolomics studies of post-mortem brain tissue in mental illness. Although this is only a small study, the findings are in agreement with several previous studies looking at specific markers and in respect of some markers with studies going back many years. The study has highlighted readily available markers which could be quantified in physiological fluids for the purpose of diagnosis or the monitoring of treatment.

## Figures and Tables

**Table 1:** Summary information for the different groups of brain samples.

Group	Number	Male	Age range	Mean age ± RSD	Female	Age range	Mean age ± RSD
Control	21	18	26–74	47.4 ± 29.5	3	42–60	50.7 ± 17.8
Schizophrenic	11	10	25–69	44.4 ± 34.7	1	40	–
Bipolar	6	1	48	–	5	39–57	45.6 ± 16.2
Depressive	7	4	24–74	47.5 ± 49.1	3	20–57	60.3 ± 33.7
Diabetic	8	8	20–69	44.9 ± 35.2	0	–	–

**Table 2:** Metabolites with high impact on the model separating controls from SDB brains (18 control/18 SDB). ^⁎^Matches retention time of standard. ^⁎⁎^Application of the Benjamini–Hochberg procedure [31] with a Q value of 0.1 indicates that the critical threshold for a regarding a P value as being significant is > 0.05. ^⁎⁎⁎^Retention time does not match that of the standard. N = negative ion P = positive ion.

m/z	Rt min.	Metabolite	VIP	P value	Ratio SDB/C
N 130.087	11.2	^⁎^Leucine/isoleucine	8.3	0.0041	1.34
N 96.9698	13.6	^⁎⁎^Orthophosphate (carbonic acid adduct of chloride)	7.5	0.0050	1.17
N 116.072	12.9	^⁎^Valine	5.1	0.0008	1.36
P 116.071	13.2	^⁎^Proline	4.2	0.0021	1.34
N 135.03	10.6	^⁎⁎^Threonic acid isomer	4.3	0.0656	1.09
N 164.072	10.4	^⁎^Phenylalanine	4.0	0.0050	1.31
N 102.056	15.9	^⁎^GABA	3.2	0.0050	0.854
N 88.0404	15.2	^⁎^Sarcosine	3.1	0.0730	1.12
N 118.051	14.8	^⁎^Homoserine	3.1	0.0140	1.51
N 267.074	11.3	^⁎^Inosine	3.0	0.0340	0.81
N 148.044	11.8	^⁎^Methionine	2.8	0.0038	1.30
N 181.072	14.3	^⁎^Sorbitol/mannitol/iditol/dulcitol	2.6	0.0022	1.99
P 258.11	14.9	^⁎^sn-glycero-3-Phosphocholine	2.2	0.0415	1.61
N 273.039	15.7	Deoxy sedoheptulose phosphate	1.9	0.0020	0.5870
N 180.067	13.4	^⁎^Tyrosine	1.7	0.0170	1.23
N 121.051	12.1	^⁎^Erythritol/threitol	1.5	0.0011	1.57
N 241.012	17.4	D-myo-Inositol 1,2-cyclic phosphate	1.3	0.0060	0.580
N 239.115	16.6	^⁎⁎^Homocarnosine isomer (anserine)	1.4	0.0037	0.622
N 303.084	17.2	^⁎^N-Acetyl-aspartyl-glutamate	1.3	0.0327	0.533
N 203.083	12.0	^⁎^Tryptophan	1.3	0.0250	1.21
N 195.051	14.4	^⁎^Gluconic acid	1.3	0.0011	2.20
N 215.033	13.7	Hexose (chloride adduct)	1.1	0.0019	2.19
N 209.067	14.4	^⁎^Sedoheptulose	1.0	0.0006	1.74
P 146.092	15.6	4-Guanidinobutanoate	1.0	0.00021	0.713

**Table 3:** Important metabolites defining the sub-group of nine SDB brains shown in Fig. 4. ^⁎^Matches retention time of standard. ^⁎⁎^Does not match the retention time of the standard therefore is an isomer of the named compound.

m/z	Rt min	Metabolite	P value	Ratio	VIP
P 227.114	10.3	^⁎⁎^Carnosine isomer	0.0020	8.34	1.70
N 145.014	6.3	^⁎⁎^Oxoglutarate isomer	0.0310	3.91	1.35
N 195.051	14.4	^⁎^Gluconic acid	< 0.001	3.30	2.32
N 159.103	4.9	Ethyl-hydroxyhexanoate	0.0020	2.95	1.35
N 181.072	14.3	^⁎^Sorbitol/mannitol/iditol/dulcitol	< 0.001	2.94	2.22
N 151.061	13.2	^⁎^Xylitol/ribitol/arabinotol	< 0.001	2.38	1.56
N 209.067	14.4	^⁎^Sedoheptulose	< 0.001	2.34	2.20
N 252.088	8.1	N-Acetylvanilalanine	< 0.001	2.06	2.15
N 121.051	12.1	^⁎^Erythritol/threitol	< 0.001	2.00	1.65
N 164.072	10.4	^⁎^Phenylalanine	< 0.001	1.88	1.80
N 178.072	12.9	^⁎^Glucosamine	0.0010	1.88	1.48
N 130.087	11.2	^⁎^Leucine	< 0.001	1.66	1.54
N 202.109	5.5	^⁎⁎^O-Acetylcarnitine isomer	< 0.001	1.60	2.13
P 116.072	12.9	^⁎^Valine	< 0.001	1.58	1.62
N 103.004	16.1	^⁎^Malonate	0.0010	1.56	1.68
P 161.107	10.4	Tryptamine	0.0010	1.55	1.39
P 169.097	11.3	^⁎^Pyridoxamine	0.0010	1.53	1.34
N 114.056	13.2	^⁎^Proline	0.002	1.52	1.99
N 180.067	13.4	^⁎^Tyrosine	0.001	1.44	1.97
P 230.151	24.2	Gamma-Aminobutyryl-lysine	0.0280	1.42	1.62
N 220.083	12.3	^⁎^N-Acetyl-D-glucosamine	< 0.001	1.40	1.99
P 104.071	15.9	^⁎^4-Aminobutanoate	< 0.001	0.78	1.40
P 284.099	13.0	^⁎^Guanosine	< 0.001	0.58	1.63
N 273.039	15.7	1-Deoxy-D-altro-heptulose 7-phosphate	0.0020	0.49	1.47
N 171.007	15.4	^⁎^Glycerol 3-phosphate	0.0040	0.38	1.88
N 231.099	16.8	N2-Succinyl-L-ornithine	0.0110	0.36	2.07
P 248.024	10.8	Norepinephrine sulfate	< 0.001	0.32	1.48
P 305.098	17.2	^⁎^N-Acetyl-aspartyl-glutamate	0.0030	0.27	2.52
P 277.031	12.6	^⁎⁎^Phospho-gluconate isomer	0.0030	0.06	1.92

**Table 4:** Marker compounds used in the OPLSDA model shown in Fig. 5.

m/z	Rt (min)	Metabolite	P value⁎	Ratio ⁎SDB/Control	VIP
116.072	12.9	L-Valine	0.00026	1.39	1.55
104.071	15.9	4-Aminobutanoate	0.0069	0.87	1.43
162.112	13.7	L-Carnitine	0.680	0.96	0.82
204.123	11.4	O-Acetylcarnitine	0.081	1.26	0.75
87.0087	8.3	Pyruvate	0.063	1.26	0.41
175.025	14.6	Ascorbate	0.85	1.03	0.37

⁎ For the 38 samples in this model.

**Table 5:** Summary of the results obtained by removing the subsets B, S, D, and DI and using them as prediction sets.

Samples removed	R^2^Y (cum) /Q^2^ (cum) for new model	Correctly classified	Incorrectly classified
B1–B6	0.721/0.596	B2–B6	B1 as control
S3–S5, S7–S11	0.706/0.601	All	
D1_D5, D6	0.623/0.526	D2–5, D7	D1 as control
DI1–DI7	0.739/0.619	DI2-DI7	DI1 as control

**Table 6:** Marker compounds used in the OPLSDA model shown in Fig. 6.

m/z	Rt (min)	Metabolite	P value⁎	Ratio ⁎SDBDI /Control	VIP
104.071	15.9	4-Aminobutanoate	0.023	0.83	1.64
116.072	12.9	L-Valine	0.0022	1.30	1.32
162.112	13.7	L-Carnitine	0.84	1.01	0.76
204.123	11.4	O-Acetylcarnitine	0.023	1.33	0.71
175.025	14.6	Ascorbate	0.834	1.04	0.50
87.0087	8.3	Pyruvate	0.063	1.26	0.48

⁎ For the 45 samples used in the model.

**Table 7:** The amounts of sugar alcohols and gluconic acid in post-mortem brain samples.

Sugar	SDB + DI (RSD) μg/g	SDB (RSD) μg/g	Control (RSD) μg/g	SDB + DI/Control ratio (P value)	SDB/Control ratio (P value)
Sorbitol	22.7 (± 56.4)	22.3 (± 56.4)	13.5 (± 57.0)	1.67 (0.0079)	1.65 (0.0015)
Gluconic acid	3.96 (± 55.8)	4.2 (55.6)	1.96 (± 40.7)	1.96 (0.0006)	2.06 (0.0012)
Ribitol	9.8 (± 21.8)	10.3 (20.2)	9.3 (± 25.9)	1.06 (0.18)	1.11 (0.06)
Arabinotol	7.9 (± 26.6)	8.0 (26.6)	8.5 (± 34.1)	0.93 (0.37)	0.94 (0.49)
Xylitol	4.1 (± 28.3)	4.3 (28.3)	7.1 (± 57.8)	0.58 (0.0042)	0.61 (0.006)
Erythritol	15.1 (± 23.8)	15.1 (23.8)	12.9 (± 34.9)	1.17 (0.028)	1.17 (0.056)

## Appendix A Supplementary data

Supplementary material Image 1
