# Supplementary material for: Metabolomic Profiling of Post-Mortem Brain Reveals Changes in Amino Acid and Glucose Metabolism in Mental Illness Compared with Controls
Source: Comput Struct Biotechnol J. 2016 Feb 26;14:106–16. doi: 10.1016/j.csbj.2016.02.003 (PMC4813093; doi:10.1016/j.csbj.2016.02.003)
Supplement: Supplementary file 1 — Supplementary material [file mmc1.docx]

**Figure A1** Permutations (n=999) plot for the OPLS-DA model shown in figure 2. The plot shows, for a selected Y-variable, on the vertical axis the values of R2 and Q2 for the original model (far to the right) and of the Y-permuted models further to the left. The horizontal axis shows the correlation between the permuted Y-vectors and the original Y-vector for the selected Y. The original Y has the correlation 1.0 with itself, defining the high point on the horizontal axis. The criteria for validity are: All blue Q2-values to the left are lower than the original points to the right or regression line of the Q2-points intersects the vertical axis (on the left) at, or below zero. Note that the R2-values always show some degree of optimism. However, when all green R2-values to the left are lower than the original point to the right, this is also an indication for the validity of the original model.


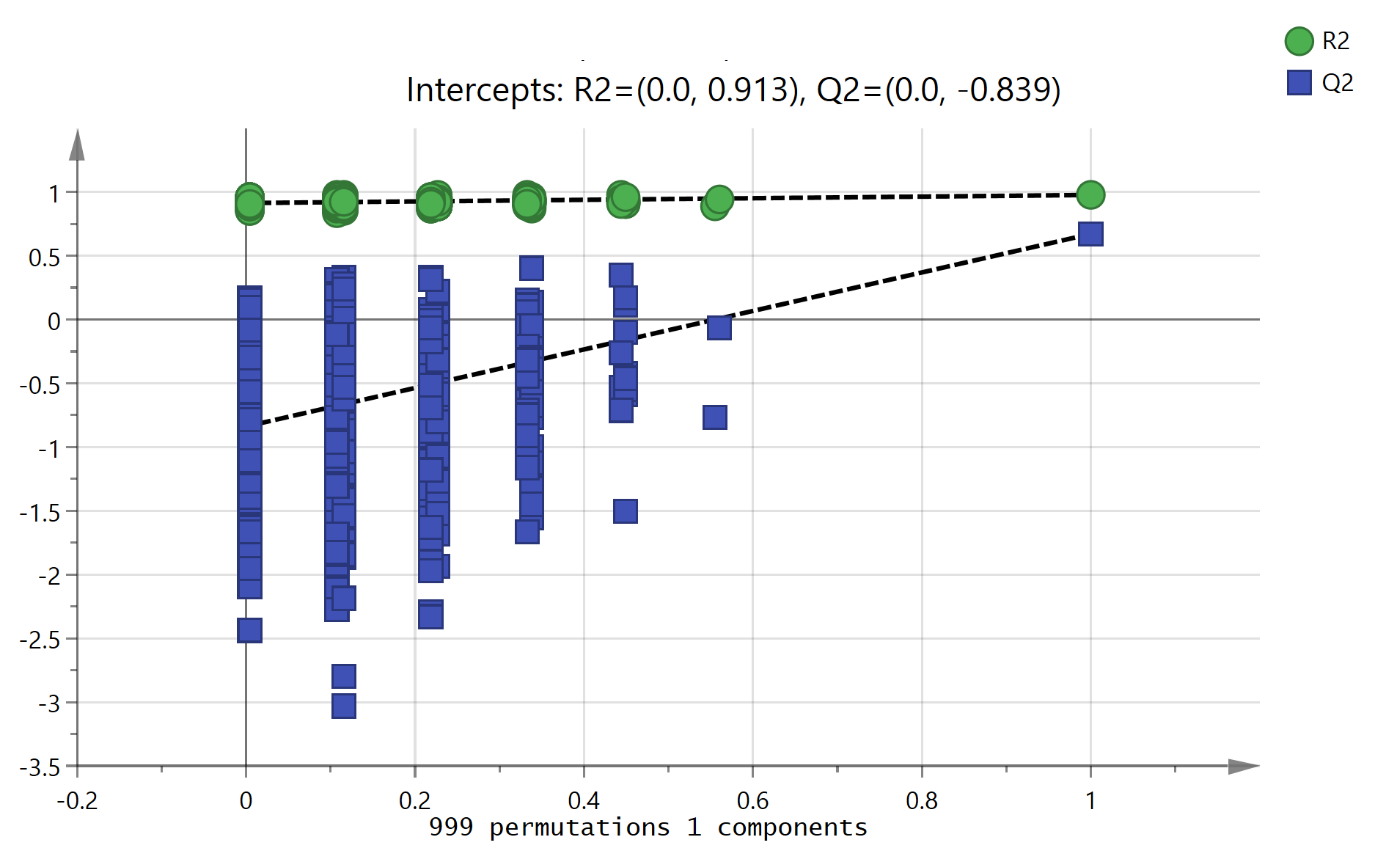


**Figure A2** OPLS correlation of brain metabolite profiles to the age of the subjects (R2X (cum) 0.706), R2Y (cum) 0.979, Q2 (cum) 0.476).


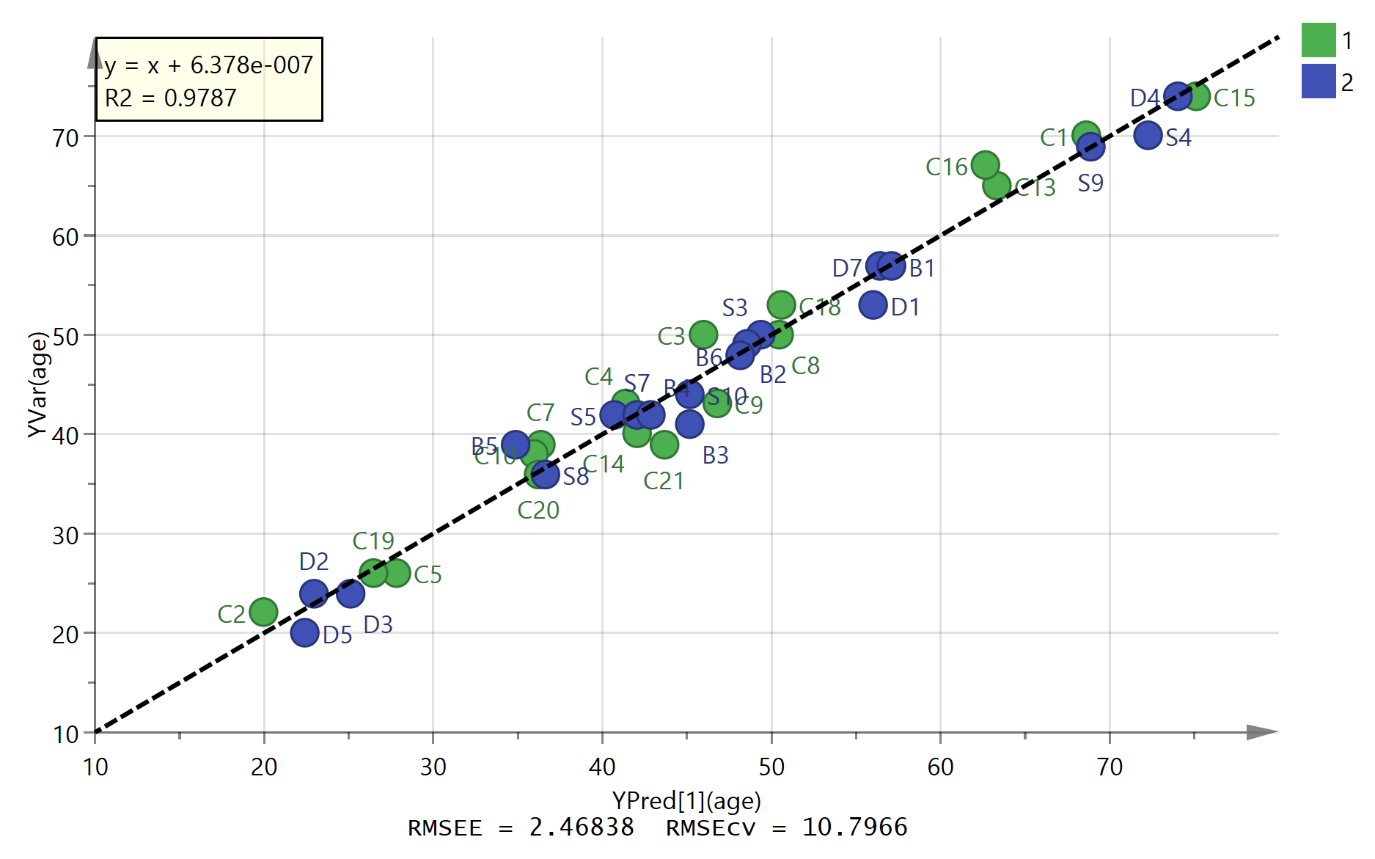


**Figure A3** Permutations (n=999) plot for the OPLS-DA model shown in figure 3.


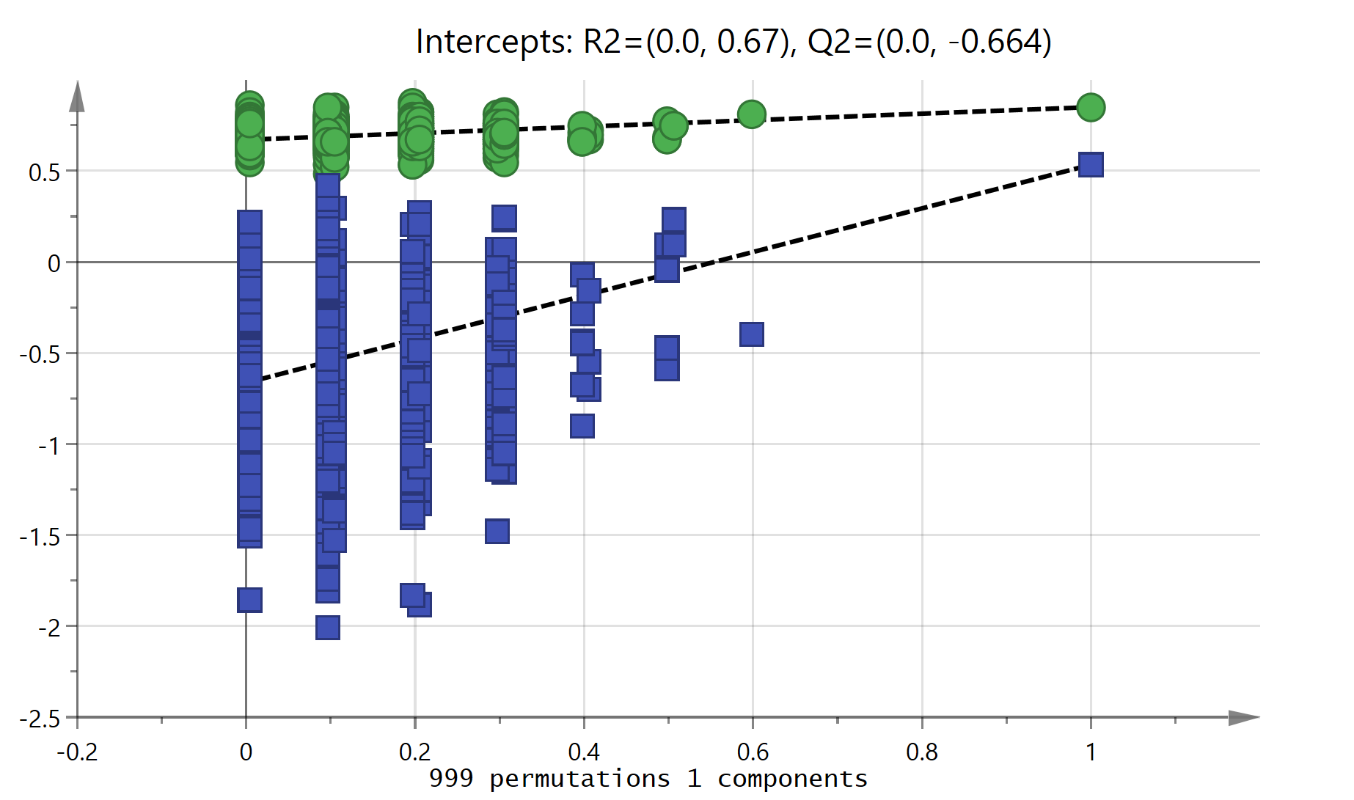


**Figure A4** HCA plot corresponding to figure 4.


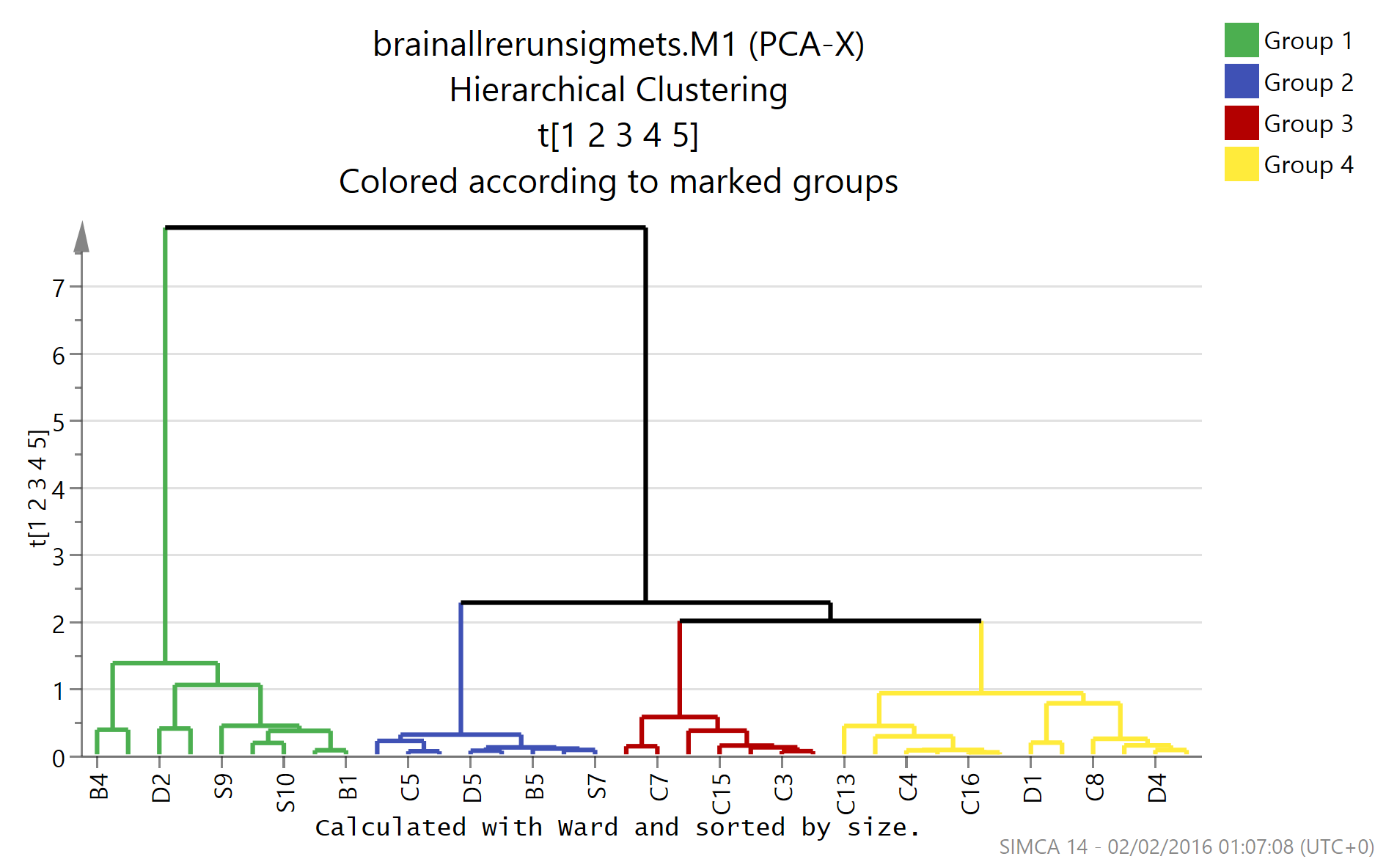


**Figure A5** Permutations (n=999) plot for the OPLS-DA model shown in figure 5.


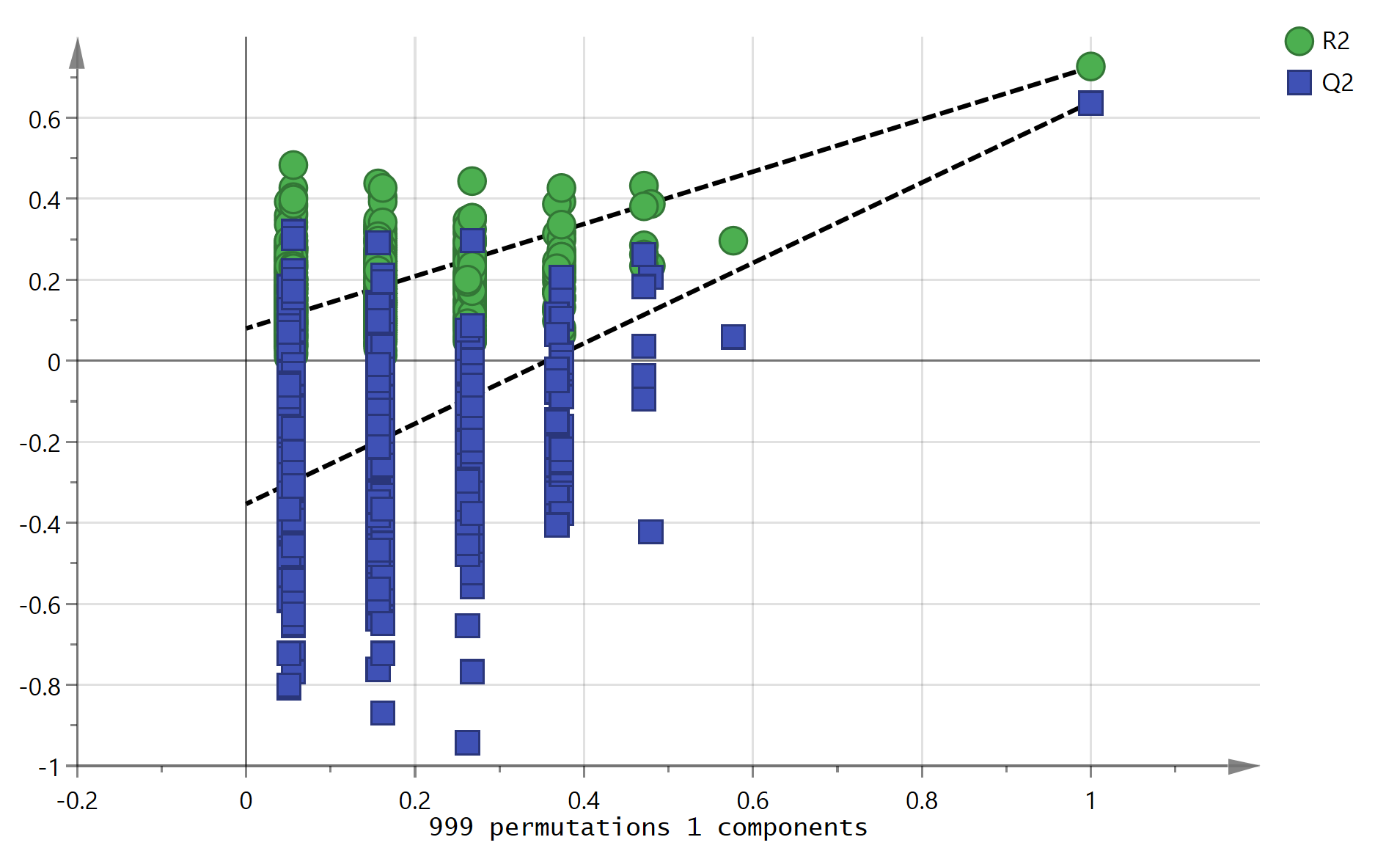


**Figure A6** Permutations (n=999) plot for the OPLS-DA model shown in figure 6.


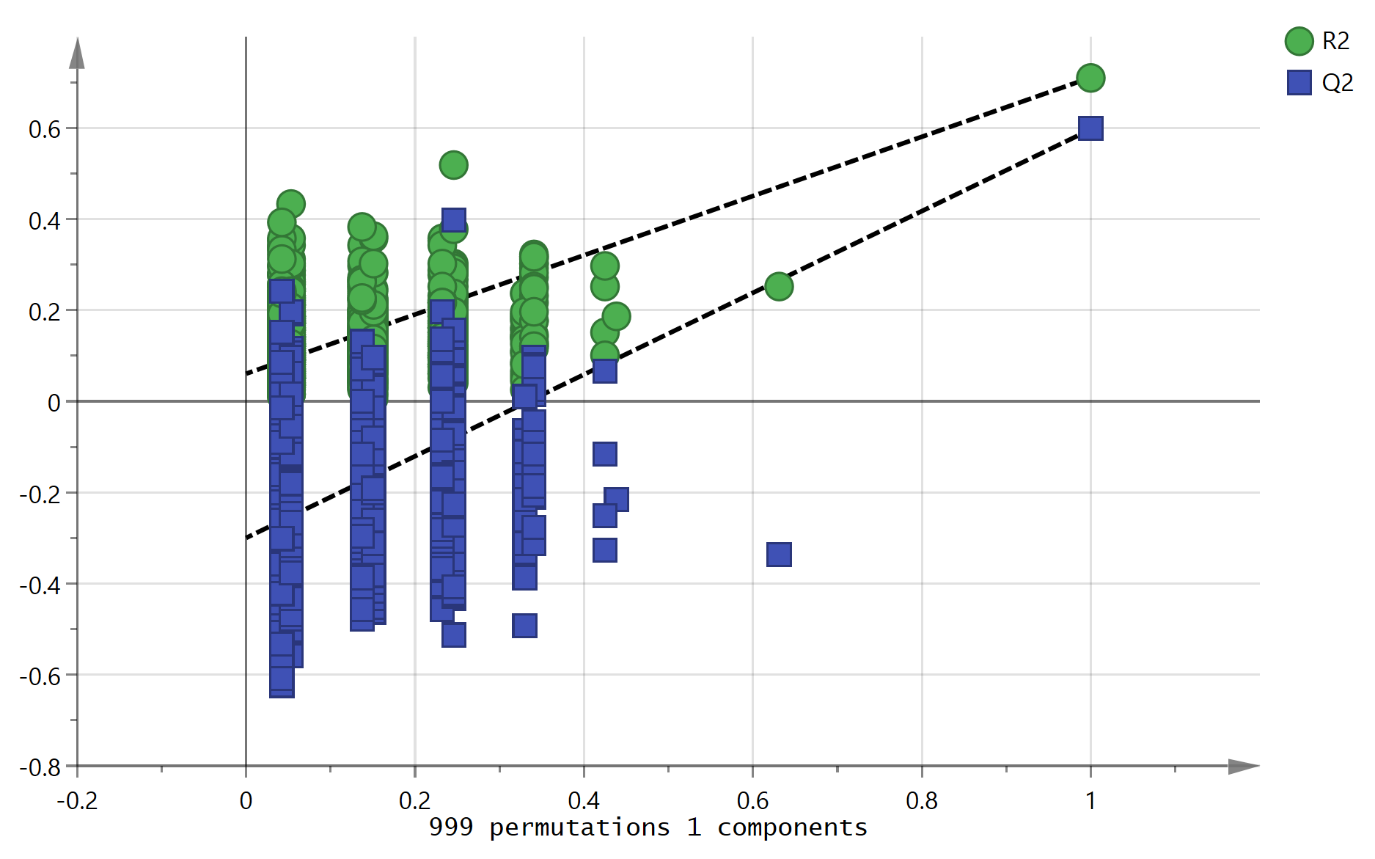


**Figure A7** Separation of SDB + DI brain samples and controls on the basis of the GC-MS analysis of sugar alcohols.


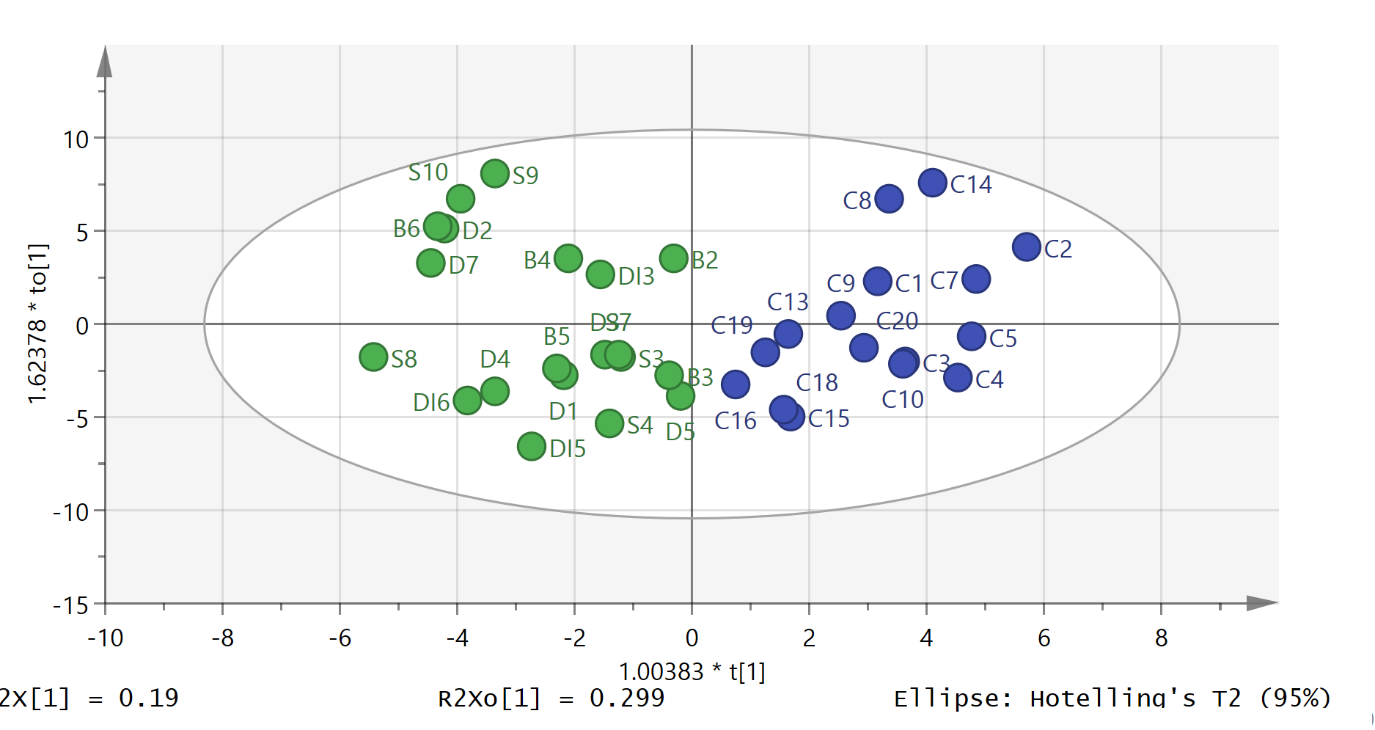


**Figure A8** Cross validation plot for figure 7.


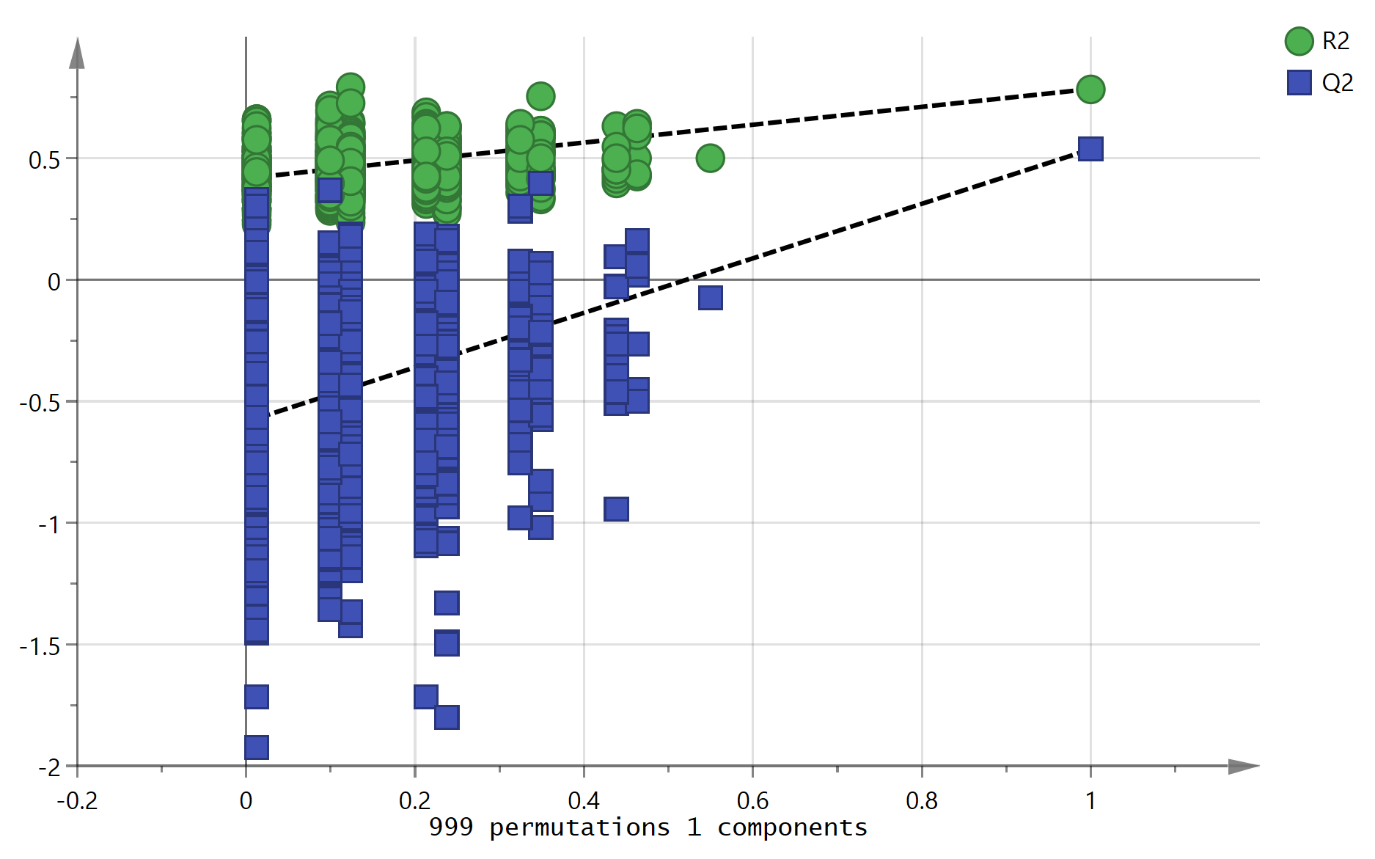


**Figure A 9** Extracted ion trace showing ascorbic acid in C5 and S7 in comparison with a standard.





**Figure A 10** Extracted ion trace showing valine in C5 and S7 in comparison with a standard.





**Figure A 11** Extracted ion trace showing GABA in C5 and S7 in comparison with a standard.

**

**

**Figure A 12** Extracted ion trace showing acetyl carnitine in C5 and S7 in comparison with a standard.

**

**

**Figure A 13** Extracted ion trace showing carnitine in C5 and S7 in comparison with a standard.

**

**

**Figure A 14** Extracted ion trace showing pyruvate in C5 and S7 in comparison with a standard.

**

**

**Table A1** Sample details C = control , D = depression, S= schizophrenic, B = bipolar, DI=diabetic

| **LC-MS Running Order** | **Pathology** | **Brain section** | **Age** | **Sex** | **Year**  **Of Collection** |
| --- | --- | --- | --- | --- | --- |
| C1 | Pulmonary embolism. Deep vein thrombosis. | Frontal convexity and frontal parasagittal | 70 | M | 2008 |
| S1 | Suspension by a ligature. | Frontal convexity and frontal parasagittal | 25 | M | 2012 |
| D1 | Suspension by a ligature. | Frontal convexity and frontal parasagittal | 53 | M | 2008 |
| D2 | Acute pulmonary oedema. | Frontal convexity and frontal parasagittal | 24 | M | 2005 |
| S2 | Intra-cerebral haemorrhage. Ruptured intra-cerebral artery. Hypertension | Frontal convexity and frontal parasagittal | 40 | F | 2009 |
| DI1 | Diabetic ketoacidosis and morphine toxicity. Ischaemic heart disease. | Frontal convexity and frontal parasagittal | 65 | M | 2010 |
| C2 | Bilateral pulmonary embolism. Deep vein thrombosis. Combined effects of hypertensive heart disease and ischaemic heart | Frontal convexity and frontal parasagittal | 22 | M | 2008 |
| S3 | Acute combined morphine, methadone, diazepam and alcohol toxicity. | Frontal convexity and frontal parasagittal | 50 | M | 2008 |
| B1 | Combined effects of chronic alcoholism and lithium use. | Frontal convexity and frontal parasagittal | 57 | F | 2005 |
| D3 | Suspension by ligature | Frontal convexity and frontal parasagittal | 24 | F | 2008 |
| C3 | Ischaemic heart disease. | Frontal convexity and frontal parasagittal | 50 | M | 2010 |
| C4 | Sudden cardiac death. | Frontal convexity and frontal parasagittal | 43 | M | 2010 |
| DI2 | Ischaemic heart disease. Coronary artery atherosclerosis. Diabetes mellitus. | Frontal convexity and frontal parasagittal | 46 | M | 2008 |
| C5 | NA | Frontal convexity and frontal parasagittal | 26 | M | 2009 |
| S4 | Ischaemic heart disease. Severe coronary artery disease. Atherosclerosis. COPD. MS | Frontal convexity and frontal parasagittal | 70 | M | 2007 |
| C6 | Multiple injuries | Frontal convexity and frontal parasagittal | 46 | M | 2009 |
| C7 | NA | Frontal convexity and frontal parasagittal | 39 | M | 2010 |
| DI3 | Ischaemic & hypertensive heart disease. Type 1 diabetes. Liver cirrhosis. | Frontal convexity and frontal parasagittal | 69 | M | 2010 |
| DI4 | Hypoglycaemia. | Frontal convexity and frontal parasagittal | 38 | M | 2008 |
| D4 | Pulmonary embolism. Deep vein thrombosis. | Frontal convexity and frontal parasagittal | 74 | M | 2011 |
| C8 | Peritonitis. Bowel infarction. | Frontal convexity and frontal parasagittal | 50 | F | 2011 |
| B2 | Metastatic carcinoma of the breast. | Frontal convexity and frontal parasagittal | 49 | F | 2010 |
| S5 | Pulmonary thromboembolism. | Frontal convexity and frontal parasagittal | 42 | M | 2005 |
| C9 | Ischaemic heart disease. Coronary artery atherosclerosis. | Frontal convexity and frontal parasagittal | 43 | M | 2010 |
| C10 | NA | Frontal convexity and frontal parasagittal | 38 | M | 2010 |
| B3 | Suspension by ligature | Frontal convexity and frontal parasagittal | 41 | F | 2007 |
| C11 | Haemopericardium. Ruptured myocardial infarction. Coronary artery atherosclerosis. Hypertensive | Frontal convexity and frontal parasagittal | 60 | F | 2009 |
| S6 | Heart disease. | Frontal convexity and frontal parasagittal | 25 | M | 2012 |
| C12 | Bronchial asthma. Pulmonary congestion. | Frontal parasagittal | 42 | F | 2011 |
| S7 | Suspension by ligature. | Frontal convexity and frontal parasagittal | 42 | M | 2009 |
| C13 | Ischaemic heart disease. Coronary artery atherosclerosis. | Frontal convexity and frontal parasagittal | 65 | M | 2009 |
| B4 | Hypertensive heart disease | Frontal convexity and frontal parasagittal | 42 | F | 2006 |
| D5 | Suspension by a ligature. | Frontal convexity and frontal parasagittal | 20 | F | 2008 |
| C14 | Pulmonary thromboembolism. Deep vein thrombosis. Rupture of Achilles tendon. | Frontal convexity and frontal parasagittal | 40 | M | 2010 |
| DI5 | Combined effects of hypertensive heart disease and ischaemic heart disease. Diabetes (Type 1). | Frontal convexity and frontal parasagittal | 45 | M | 2008 |
| S8 | Unascertained pending laboratory studies. | Frontal convexity and frontal parasagittal | 36 | M | 2011 |
| C15 | Ischaemic heart disease. Coronary artery atherosclerosis. | Frontal convexity and frontal parasagittal | 74 | M | 2010 |
| D6 | Suspension by a ligature. | Frontal convexity and frontal parasagittal | 32 | M | 2009 |
| B5 | Suspension by a ligature. | Frontal convexity and frontal parasagittal | 39 | F | 2011 |
| C16 | Ischaemic heart disease. Coronary artery thrombosis. Coronary artery atherosclerosis. | Frontal convexity and frontal parasagittal | 67 | M | 2009 |
| DI6 | Hypertensive heart disease. Metastatic testicular tumour. COPD. | Frontal convexity and frontal parasagittal | 38 | M | 2007 |
| S9 | Hypertensive heart disease. Chronic obstructive pulmonary disease. | Frontal convexity and frontal parasagittal | 69 | M | 2009 |
| C17 | Myocardial re-infarction. Previous myocardial infarcts. Coronary atherosclerosis. Hypertensive heart disease. | Frontal convexity and frontal parasagittal | 48 | M | 2010 |
| DI7 | Hypoglycaemia. | Frontal convexity and frontal parasagittal | 38 | M | 2008 |
| S10 | Hypertensive heart disease. Hypertension. | Frontal convexity and frontal parasagittal | 44 | M | 2006 |
| B6 | Bronchopneumonia. | Frontal convexity and frontal parasagittal | 48 | M | 2011 |
| C18 | Ischaemic heart disease. Coronary artery atherosclerosis. | Frontal convexity and frontal parasagittal | 53 | M | 2006 |
| DI8 | Hyperglycaemic coma. | Frontal convexity and frontal parasagittal | 20 | M | 2006 |
| C19 | NA | Frontal convexity and frontal parasagittal | 26 | M | 2009 |
| D7 | Pulmonary thromboembolism. | Frontal convexity and frontal parasagittal | 57 | F | 2008 |
| S11 | External asphyxia by inhalation of helium. | Frontal convexity and frontal parasagittal | 41 | M | 2008 |
| C20 | Cardiomegaly. Ischaemic heart disease. | Frontal convexity and frontal parasagittal | 36 | M | 2010 |
| C21 | NA | Frontal convexity and frontal parasagittal | 39 | M | 2010 |

**Table A2** Summary of univariate differences with a P value < 0.05** between control and SDB samples used to build the OPLSDA model shown in figure 1.** Application of the * Indicates that the retention time matches that of an authentic standard thus identification of MSI level 1. Application of the Benjamini-Hochberg procedure [31] with a Q value of 0.1 indicates that the critical threshold for a regarding a P value as being significant is >0.05.

| row ID | row m/z | row retention time | Molecular formula | Name | Pvalue | Ratio |
| --- | --- | --- | --- | --- | --- | --- |
| **Neurotransmitter metabolism** | | | | | | |
| 4191 | 146.0924 | 15.6 | C5H11N3O2 | 4-Guanidinobutanoate | 0.0002 | 0.714 |
| 4139 | 104.0706 | 15.9 | C4H9NO2 | 4-Aminobutanoate* | 0.005 | 0.854 |
| 767 | 248.0238 | 9.1 | C8H11NO6S | Norepinephrinesulfate | 0.008 | 0.489 |
| 776 | 248.0238 | 10.8 | C8H11NO6S | Norepinephrinesulfate | 0.010 | 0.526 |
| 25 | 164.072 | 10.4 | C9H11NO2 | Phenylalanine* | 0.005 | 1.312 |
| 3337 | 260.0237 | 15.6 | C9H11NSO6 | tyrosine sulfate | 0.009 | 1.319 |
| 50 | 180.0669 | 13.4 | C9H11NO3 | Tyrosine* | 0.017 | 1.228 |
| 368 | 252.0882 | 8.1 | C12H15NO5 | N-Acetylvanilalanine | 0.017 | 1.455 |
| 89 | 146.0461 | 11.0 | C5H9NO4 | Glutamate* | 0.021 | 1.127 |
| 6505 | 189.087 | 10.9 | C7H12N2O4 | N-Acetylglutamine | 0.045 | 1.233 |
| 67 | 203.083 | 12.0 | C11H12N2O2 | Tryptophan* | 0.025 | 1.205 |
| 4366 | 161.1073 | 10.4 | C10H12N2 | Tryptamine | 0.030 | 1.259 |
| 4307 | 305.0978 | 17.2 | C11H16N2O8 | N-Acetyl-aspartyl-glutamate* | 0.029 | 0.489 |
| 2248 | 207.0778 | 9.4 | C10H12N2O3 | Kynurenine* | 0.010 | 1.436 |
| 6253 | 165.1023 | 6.6 | C9H12N2O | Kynuramine | 0.048 | 1.462 |
| **Neutral lipophilic amino acids and metabolites** | | | | | | |
| 20 | 116.0718 | 12.9 | C5H11NO2 | L-Valine* | 0.0008 | 1.358 |
| 48 | 114.0561 | 13.2 | C5H9NO2 | L-Proline* | 0.002 | 1.341 |
| 46 | 148.044 | 11.8 | C5H11NO2S | L-Methionine* | 0.004 | 1.297 |
| 11 | 130.0875 | 11.2 | C6H13NO2 | L-Leucine/isoleucine* | 0.004 | 1.344 |
| 7122 | 174.1125 | 7.1 | C8H15NO3 | N-Acetyl-L-leucine | 0.017 | 2.314 |
| 6004 | 204.0867 | 14.1 | C8H13NO5 | N2-Acetyl-L-aminoadipate | 0.003 | 0.772 |
| **Polar amino acids** | | | | | | |
| 2284 | 104.0354 | 16.1 | C3H7NO3 | L-Serine* | 0.021 | 1.439 |
| 1034 | 118.0511 | 14.8 | C4H9NO3 | L-Threonine* | 0.014 | 1.523 |
| 4160 | 90.05499 | 15.2 | C3H7NO2 | Sarcosine* | 0.023 | 1.093 |
| 2299 | 231.099 | 16.8 | C9H16N2O5 | N2-Succinyl-L-ornithine | 0.017 | 0.414 |
| **Sugar metabolism** | | | | | | |
| 172 | 209.067 | 14.4 | C7H14O7 | Sedoheptulose* | 0.0006 | 1.741 |
| 200 | 195.0513 | 14.4 | C6H12O7 | Gluconic/gulonic acid | 0.001 | 2.203 |
| 154 | 121.0507 | 12.1 | C4H10O4 | Erythritol/threitol | 0.001 | 1.574 |
| 266 | 215.0332 | 13.7 | C6H11O6Cl | hexose chloride adduct | 0.002 | 2.186 |
| 85 | 181.072 | 14.3 | C6H14O6 | Sorbitol/mannitol/adonitol/dulcitol | 0.002 | 1.988 |
| 57 | 241.0123 | 17.4 | C6H11O8P | D-myo-Inositol 1,2-cyclic phosphate | 0.006 | 0.580 |
| 160 | 421.0761 | 17.4 | C12H23O14P | Trehalose phosphate | 0.009 | 0.623 |
| 169 | 209.0306 | 16.0 | C6H10O8 | Glucarate/galactarate | 0.013 | 1.312 |
| 90 | 151.0614 | 13.2 | C5H12O5 | Xylitol/ribitol/arabinitol | 0.017 | 1.144 |
| 1896 | 341.1095 | 15.6 | C12H22O11 | Sucrose | 0.026 | 0.601 |
| 42 | 273.0385 | 15.7 | C7H15O9P | 1-Deoxy-D-altro-heptulose 7-phosphate | 0.002 | 0.587 |
| 3327 | 177.0406 | 15.6 | C6H10O6 | D-Glucono-1,5-lactone | 0.049 | 2.745 |
| **Amino sugar metabolism** | | | | | | |
| 265 | 290.0887 | 13.6 | C11H17NO8 | Anhydro-alpha-N-acetylneuraminic acid | 0.0005 | 0.770 |
| 1001 | 204.088 | 11.4 | C8H15NO5 | N-Acetyl-D-fucosamine | 0.042 | 1.503 |
| 600 | 220.083 | 12.3 | C8H15NO6 | N-Acetyl-D-glucosamine | 0.0005 | 1.244 |
| 3228 | 178.0724 | 12.9 | C6H13NO5 | D-Glucosamine | 0.0008 | 1.845 |
| 7702 | 221.1132 | 14.1 | C8H16N2O5 | N-Acetyl-beta-D-glucosaminylamine | 0.046 | 0.823 |
| 4642 | 260.0528 | 15.8 | C6H14NO8P | D-Glucosamine 6-phosphate | 0.032 | 1.566 |
| **Glycolysis and Krebs Cycle** | | | | | | |
| 273 | 103.0038 | 16.1 | C3H4O4 | Malonate* | 0.007 | 1.298 |
| 183 | 173.0094 | 12.1 | C6H6O6 | cis-Aconitate isomer | 0.011 | 1.393 |
| 5302 | 277.0311 | 12.6 | C6H13O10P | 6-Phospho-D-gluconate isomer | 0.015 | 0.256 |
| 225 | 173.0093 | 18.3 | C6H6O6 | cis-Aconitate* | 0.007 | 0.644 |
| 3601 | 145.0144 | 6.3 | C5H6O5 | 2-Oxoglutarate isomer | 0.019 | 3.084 |
| **Carnitines** | | | | | | |
| 122 | 202.1089 | 5.5 | C9H18NO4 | O-Acetylcarnitine isomer | 0.007 | 1.486 |
| 7137 | 442.3526 | 5.1 | C25H47NO5 | 3-Hydroxy-11Z-octadecenoylcarnitine | 0.003 | 0.448 |
| 5789 | 290.1596 | 12.5 | C13H23NO6 | 3-Methylglutarylcarnitine | 0.011 | 0.684 |
| 281 | 368.2813 | 4.0 | C21H39NO4 | cis-5-Tetradecenoylcarnitine | 0.017 | 0.493 |
| 957 | 202.1089 | 11.4 | C9H18NO4 | O-Acetylcarnitine* | 0.041 | 1.361 |
| 5741 | 330.2637 | 4.2 | C18H35NO4 | undecanoylcarnitine | 0.047 | 1.903 |
| **Purine and Pyrimidine Metabolism** | | | | | | |
| 2485 | 347.0404 | 15.9 | C10H13N4O8P | IMP* | 0.051 | 0.678 |
| 4138 | 264.1094 | 11.4 | C11H15N5O3 | N6-Methyl-2'-deoxyadenosine | 0.041 | 1.480 |
| 4221 | 284.0988 | 13.0 | C10H13N5O5 | Guanosine* | 0.030 | 0.773 |
| 4152 | 269.0879 | 11.3 | C9H16O9 | Inosine* | 0.019 | 0.804 |
| 192 | 167.0213 | 13.0 | C5H4N4O3 | Urate* | 0.027 | 1.555 |
| 4480 | 152.0566 | 12.8 | C5H5N5O | Guanine | 0.026 | 0.702 |
| 596 | 113.0358 | 7.8 | C4H6N2O2 | 5,6-Dihydrouracil | 0.009 | 1.474 |
| 2167 | 245.0782 | 10.4 | C9H14N2O6 | 5-6-Dihydrouridine | 0.009 | 1.442 |
| 251 | 255.0991 | 9.4 | C11H16N2O5 | Methyluridine | 0.008 | 1.399 |
| 4997 | 286.1032 | 8.5 | C11H15N3O6 | N4-Acetylcytidine | 0.030 | 1.436 |
| **Glycerolipid metabolism** | | | | | | |
| 1233 | 885.5504 | 3.9 | C47H83O13P | [PI (18:0/20:4)] 1-octadecanoyl-2-(5Z,8Z,11Z,14Z-eicosatetraenoyl)-sn-glycero-3-phospho-(1'-myo-inositol) | 0.001 | 1.714 |
| 6999 | 820.6219 | 4.1 | C48H86NO7P | PC(22:4(7Z,10Z,13Z,16Z)/P-18:1(11Z)) | 0.040 | 0.645 |
| 4532 | 744.5905 | 4.3 | C42H82NO7P | 1-Hexadecanoyl-2-(9Z-octadecenoyl)-sn-glycero-3-phosphonocholine | 0.006 | 0.561 |
| 4229 | 786.6012 | 4.2 | C44H84NO8P | [PC (18:1/18:1)] 1-(9Z-octadecenoyl)-2-(9Z-octadecenoyl)-sn-glycero-3-phosphocholine | 0.008 | 0.870 |
| 7001 | 734.5701 | 4.3 | C40H80NO8P | [PC (16:0/16:0)] 1-hexadecanoyl-2-hexadecanoyl-sn-glycero-3-phosphocholine | 0.0009 | 1.327 |
| 4555 | 706.5384 | 4.3 | C38H76NO8P | [PC (15:0/15:0)] 1,2-dipentadecanoyl-sn-glycero-3-phosphocholine | 0.001 | 1.239 |
| 337 | 597.3056 | 4.5 | C27H51O12P | [PI (18:0)] 1-(9Z-octadecenoyl)-sn-glycero-3-phospho-(1'-myo-inositol) | 0.008 | 0.768 |
| 6550 | 720.5552 | 4.4 | C39H78NO8P | [PC (15:0/16:0)] 1-pentadecanoyl-2-hexadecanoyl-sn-glycero-3-phosphocholine | 0.012 | 1.507 |
| 4599 | 796.5856 | 4.1 | C45H82NO8P | PE(18:0/22:4(7Z,10Z,13Z,16Z)) | 0.018 | 1.130 |
| 69 | 509.2892 | 4.0 | C24H47O9P | [PG (18:0)] 1-(9E-octadecenoyl)-sn-glycero-3-phospho-(1'-sn-glycerol) | 0.024 | 0.804 |
| 4554 | 511.3032 | 4.0 | C24H47O9P | [PG (18:0)] 1-(9E-octadecenoyl)-sn-glycero-3-phospho-(1'-sn-glycerol) | 0.032 | 0.817 |
| 4533 | 770.6065 | 4.2 | C44H84NO7P | PC(18:1(11Z)/P-18:1(11Z)) | 0.037 | 0.513 |
| 4993 | 536.3348 | 4.4 | C26H50NO8P | [PC acetyl(16:0)] 1-(9Z-hexadecenoyl)-2-acetyl-sn-glycero-3-phosphocholine | 0.043 | 0.585 |
| 377 | 483.2738 | 4.0 | C22H45O9P | [PG (16:0)] 1-hexadecanoyl-sn-glycero-3-phospho-(1'-sn-glycerol) | 0.048 | 0.809 |
| 4744 | 258.11 | 14.9 | C8H20NO6P | sn-glycero-3-Phosphocholine | 0.042 | 1.606 |
| 4471 | 247.0576 | 13.2 | C6H15O8P | Glycerophosphoglycerol | 0.001 | 0.793 |
| 128 | 171.0066 | 14.9 | C3H9O6P | sn-Glycerol 3-phosphate | 0.034 | 1.684 |
| **Miscellaneous** | | | | | | |
| 59 | 239.1154 | 16.6 | C10H16N4O3 | Homocarnosine | 0.004 | 0.622 |
| 7071 | 227.1139 | 10.3 | C9H14N4O3 | Carnosine isomer (probably anserine) | 0.0009 | 5.168 |
| 4239 | 170.0812 | 8.1 | C8H11NO3 | Pyridoxine | 0.005 | 1.678 |
| 5958 | 169.0971 | 11.3 | C8H12N2O2 | Pyridoxamine | 0.049 | 1.245 |
| 3 | 96.96983 | 13.6 | H3O4P | Orthophosphate | 0.005 | 1.168 |
| 3930 | 157.0872 | 5.5 | C8H14O3 | [FA oxo(8:0)] 3-oxo-octanoic acid | 0.001 | 1.571 |
| 7654 | 316.2845 | 4.0 | C18H37NO3 | [SP hydrox] 6-hydroxysphing-4E-enine | 0.005 | 1.611 |
| 5445 | 176.0554 | 15.0 | C6H9NO5 | [FA amino,oxo(6:0/2:0)] 2-amino-3-oxo-hexanedioic acid | 0.008 | 0.611 |
| 1665 | 141.0923 | 4.6 | C8H14O2 | [FA (8:0)] 2Z-octenoic acid | 0.012 | 1.442 |
| 558 | 159.1029 | 4.9 | C8H16O3 | Ethyl (R)-3-hydroxyhexanoate | 0.015 | 2.012 |
| 1761 | 309.2229 | 4.2 | C22H30O | [ST ethyl,methy] 13-ethyl-11-methylene-18,19-dinorpregn-4-en-20-yn-17alpha-ol | 0.017 | 0.612 |
| 2237 | 315.2547 | 4.1 | C18H36O4 | [FA hydroxy(18:0)] 9,10-dihydroxy-octadecanoic acid | 0.018 | 1.953 |
| 479 | 171.1029 | 4.7 | C9H16O3 | 9-Oxononanoic acid | 0.019 | 1.416 |
| 7062 | 188.103 | 12.5 | C7H13N3O3 | 5-guanidino-3-methyl-2-oxo-pentanoate | 0.021 | 1.443 |
| 355 | 131.0351 | 14.0 | C5H8O4 | 2-Acetolactate | 0.024 | 1.438 |
| 7036 | 223.0747 | 4.5 | C7H14N2O4S | L-Cystathionine* | 0.026 | 3.785 |
| 279 | 131.0715 | 6.2 | C6H12O3 | 6-Hydroxyhexanoic acid | 0.027 | 1.445 |
| 526 | 230.1513 | 24.2 | C10H21N3O3 | Gamma-Aminobutyryl-lysine | 0.027 | 0.457 |
| 3230 | 351.2184 | 5.4 | C20H32O5 | [FA oxo,hydroxy(2:0)] 9-oxo-11R,15S-dihydroxy-5Z,13E-prostadienoic acid [PGE_2_] | 0.027 | 10.749 |
| 276 | 131.0351 | 11.0 | C5H8O4 | 2-Acetolactate | 0.029 | 6.808 |
| 222 | 155.0464 | 8.5 | C6H8N2O3 | 4-Imidazolone-5-propanoate | 0.030 | 2.317 |
| 1186 | 572.0808 | 13.2 | C16H25N5O14P2 | GDP-3,6-dideoxy-D-galactose | 0.033 | 0.654 |
| 981 | 145.0872 | 4.9 | C7H14O3 | [FA hydroxy(7:0)] 2-hydroxy-heptanoic acid | 0.033 | 1.333 |
| 7167 | 257.1495 | 5.6 | C12H20N2O4 | N-heptadieneoylglutamine | 0.034 | 1.661 |
| 275 | 295.2651 | 3.9 | C19H36O2 | [FA methyl(18:0)] 11R,12S-methylene-octadecanoic acid | 0.037 | 0.811 |
| 2549 | 189.0044 | 12.2 | C6H6O7 | Oxalosuccinate | 0.038 | 1.869 |
| 1676 | 335.2235 | 4.9 | C20H32O4 | Leukotriene B4 | 0.040 | 9.210 |
| 1522 | 219.015 | 12.1 | C7H8O8 | 4-Carboxy-4-hydroxy-2-oxoadipate | 0.042 | 1.752 |
| 661 | 171.0066 | 15.4 | C3H9O6P | *sn-Glycerol 3-phosphate | 0.045 | 0.598 |
| 1674 | 159.1028 | 6.4 | C8H16O3 | Ethyl (R)-3-hydroxyhexanoate | 0.047 | 1.419 |
| 4370 | 648.6294 | 4.0 | C42H81NO3 | [SP (24:0)] N-(15Z-tetracosenoyl)-sphing-4-enine | 0.050 | 0.622 |
| 3202 | 175.0978 | 5.0 | C8H16O4 | [FA hydroxy(8:0)] 6,8-dihydroxy-octanoic acid | 0.050 | 1.598 |

**Table A3** Most significant metabolite changes with the age of brain tissue.

| ID | m/z | Rt | Metabolite | P corr | VIP |
| --- | --- | --- | --- | --- | --- |
| **1** | 303.234 | 3.9 | [FA (20:4)] 5Z,8Z,11Z,14Z-eicosatetraenoic acid | -0.47 | 10.98 |
| **2** | 91.04 | 10.6 | Glycerol | -0.55 | 8.69 |
| **6** | 281.249 | 4.0 | [FA (18:0)] 9Z-octadecenoic acid | -0.45 | 7.80 |
| **12** | 327.234 | 3.9 | Docosahexaenoicacid | -0.32 | 6.99 |
| **15** | 255.233 | 4.0 | Hexadecanoic acid | -0.37 | 6.16 |
| **3** | 96.9698 | 13.6 | Orthophosphate | 0.38 | 5.43 |
| **9** | 128.035 | 10.6 | L-1-Pyrroline-3-hydroxy-5-carboxylate | -0.51 | 5.40 |
| **4141** | 116.071 | 13.2 | L-Proline | -0.41 | 4.47 |
| **28** | 253.218 | 4.0 | (9Z)-Hexadecenoic acid | -0.44 | 3.77 |
| **53** | 279.233 | 4.0 | Linoleate | -0.41 | 2.66 |
| **48** | 114.056 | 13.2 | L-Proline | -0.36 | 2.05 |
| **4744** | 258.11 | 14.9 | sn-glycero-3-Phosphocholine | 0.38 | 2.02 |
| **31** | 111.02 | 8.8 | Uracil | -0.35 | 1.90 |
| **54** | 227.202 | 4.1 | Tetradecanoic acid | -0.37 | 1.89 |
| **30** | 214.049 | 16.0 | sn-glycero-3-Phosphoethanolamine | 0.49 | 1.81 |
| **41** | 175.025 | 14.6 | Ascorbate | 0.35 | 1.56 |
| **50** | 180.067 | 13.4 | L-Tyrosine | -0.34 | 1.39 |
| **4157** | 178.072 | 10.5 | 6-methyl-H2-pterin | -0.54 | 1.12 |
| **55** | 308.099 | 13.8 | N-Acetylneuraminate | 0.37 | 0.97 |
| **101** | 165.041 | 13.1 | L-Arabinonate | -0.48 | 0.97 |
| **135** | 309.281 | 3.9 | [FA (20:0)] 11Z-eicosenoic acid | -0.45 | 0.80 |

**Table A4** Metabolites with high impact on the model separating controls from SDB brains combined with diabetic brains. * Matches retention time of standard. ** Retention time does not match that of the standard. N= negative ion P =positive ion.

| **m/z** | **Rt min** | **Metabolite** | **VIP value** |
| --- | --- | --- | --- |
| 96.9698 | 13.6 | chloride carbonic acid adduct | 8.4 |
| N130.087 | 11.2 | *L-Leucine | 7.2 |
| N174.041 | 15.0 | *N-Acetyl-L-aspartate | 4.9 |
| N116.072 | 12.9 | *L-Valine | 4.6 |
| N164.072 | 10.4 | *L-Phenylalanine | 3.9 |
| N102.056 | 15.9 | *4-Aminobutanoate | 3.1 |
| N118.051 | 14.8 | *L-Threonine | 2.8 |
| N181.072 | 14.3 | *D-Sorbitol | 2.7 |
| N267.074 | 11.3 | *Inosine | 2.6 |
| N114.056 | 13.2 | *L-Proline | 2.5 |
| N148.044 | 11.8 | *L-Methionine | 2.4 |
| P204.123 | 11.4 | *O-Acetylcarnitine | 2.4 |
| 273.039 | 15.7 | deoxysedoheptulose phosphate | 2.0 |
| *121.041 | 7.3 | *Nicotinamide | 1.8 |
| P241.129 | 16.6 | Anserine | 1.5 |
| N121.051 | 12.1 | *Erythritol | 1.5 |
| N145.014 | 13.5 | **2-Oxoglutarate | 1.5 |
| N241.012 | 17.4 | D-myo-Inositol 1,2-cyclic phosphate | 1.4 |
| N195.051 | 14.4 | *D-Gluconic acid | 1.3 |
| N209.031 | 16.0 | *D-Glucarate | 1.1 |
| 215.033 | 13.7 | hexose chloride adduct | 1.1 |
| 170.081 | 8.1 | *Pyridoxine | 1.0 |
| 209.067 | 14.4 | *Sedoheptulose | 0.9 |
| 146.092 | 15.6 | 4-Guanidinobutanoate | 0.9 |

**Table A5** Retention data for polyol isomers and acids analysed by GC-MS in EI mode.

| **Polyol** | **MW** | **Rat pHILIC** | **Rat GC** | **MW**  **Derivative** | **EIMS Main Fragments** |
| --- | --- | --- | --- | --- | --- |
| Gulonic acid | 196 |  | 20.66 | 420 | 184 (70)145(100)142(97)112(56)103(92) |
| Gluconic acid | 196 |  | 20.82 | 420 | 217 (25) 173 (56) 157(38) 155 (69) 115(100) 103(51) |
| Mannitol | 182 |  | 21.95 | 434 | 217 (25) 187(52) 157 (32) 145(65)115(100)103(57) |
| Sorbitol | 182 |  | 22.11 | 434 | 271 (18) 187 (48) 157 (34) 145 (68) 115 (100) |
| Dulcitol | 182 |  | 22.25 | 434 | 217 (19) 187(49) 155 (42) 145(50)115(100) |
| Iditol | 182 |  | 22.33 | 434 | 217 (26) 187 (33) 170 (27) 157 (34) 145 (44) 115( 100) |
| Myoinositol | 180 |  | 21.99 | 432 | 210 (37) 168 (100) 157 (39) 126 (56) 115 (66) |
| Pinitol | 194 |  | 20.80 | 404 | 182 (74) 140 (100) 87 (62) |
| Ribitol | 152 |  | 17.53 | 362 | 217 (21) 187 (9) 145(69) 115(100)103(89) |
| Arabinitol | 152 |  | 17.67 | 362 | 217 (22) 187 (39) 145(65)115(100)103 (72) |
| Xylitol | 152 |  | 18.02 | 362 | 217 (30) 187 (32) 145(68)115(100)103 (68) |
| Threitol | 122 |  | 12.88 | 290 | 217(8) 145 (74) 128 (28) 115 (100) 103 (81) |
| Erythritol | 122 |  | 12.49 | 290 | 217 145 (11) 145 (85) 128 (33) 115(94) 103 (100) |

**Table A6** Calibration curve data for sugar alcohols and gluconic acid over the range 1-16 µg with pinitol as an internal standard.

| **Metabolite** | **Equation of the line (R^2^ value)** |
| --- | --- |
| Sorbitol | Y=0.1093 x – 0.545 (0.9994) |
| Gluconic acid | Y=0.0120 x + 0.00113 (0.9660) |
| Ribitol | Y=0.1252x – 0.0475 (0.9998) |
| Arabinotol | Y=0.1228x -0.0448 (0.9991) |
| Xylitol | Y=0.1277x- 0.0454 (0.9996) |
| Erythritol | Y= 0.0450 – 0.0563 (0.9918) |
